# Origin and History of Mitochondrial DNA Lineages in Domestic Horses

**DOI:** 10.1371/journal.pone.0015311

**Published:** 2010-12-20

**Authors:** Michael Cieslak, Melanie Pruvost, Norbert Benecke, Michael Hofreiter, Arturo Morales, Monika Reissmann, Arne Ludwig

**Affiliations:** 1 Leibniz Institute for Zoo and Wildlife Research, Evolutionary Genetics, Berlin, Germany; 2 German Archaeological Institute, Berlin, Germany; 3 Max Planck Institute for Evolutionary Anthropology, Leipzig, Germany; 4 Department of Biology, The University of York, Heslington, York, United Kingdom; 5 Laboratory of Archaeozoology, Universidad Autonoma Madrid, Madrid, Spain; 6 Department of Breeding Biology and Molecular Genetics, Humboldt University Berlin, Berlin, Germany; Erasmus University Medical Center, The Netherlands

## Abstract

Domestic horses represent a genetic paradox: although they have the greatest number of maternal lineages (mtDNA) of all domestic species, their paternal lineages are extremely homogeneous on the Y-chromosome. In order to address their huge mtDNA variation and the origin and history of maternal lineages in domestic horses, we analyzed 1961 partial d-loop sequences from 207 ancient remains and 1754 modern horses. The sample set ranged from Alaska and North East Siberia to the Iberian Peninsula and from the Late Pleistocene to modern times. We found a panmictic Late Pleistocene horse population ranging from Alaska to the Pyrenees. Later, during the Early Holocene and the Copper Age, more or less separated sub-populations are indicated for the Eurasian steppe region and Iberia. Our data suggest multiple domestications and introgressions of females especially during the Iron Age. Although all Eurasian regions contributed to the genetic pedigree of modern breeds, most haplotypes had their roots in Eastern Europe and Siberia. We found 87 ancient haplotypes (Pleistocene to Mediaeval Times); 56 of these haplotypes were also observed in domestic horses, although thus far only 39 haplotypes have been confirmed to survive in modern breeds. Thus, at least seventeen haplotypes of early domestic horses have become extinct during the last 5,500 years. It is concluded that the large diversity of mtDNA lineages is not a product of animal breeding but, in fact, represents ancestral variability.

## Introduction

The domestication of animals and plants was a key innovation in the emergence of modern urban societies. Deciphering the spatial and temporal origin of the domestic species seems crucial for understanding the cultural changes of civilizations and has consequently attracted considerable scientific as well as public interest [1], [2]. Among the early domesticated animals, two species of major importance differ from all others by having been bred not mainly as food sources: the dog and the horse. Whereas the dog may arguably have been the first animal to be domesticated [3], [4 – commonly used for hunting, guard and military actions], horses attained a prominent role as animals of transport and warfare, changing societies on a continent-wide scale. As M. Levine so aptly remarked: *“Before the development of firearms, the horse was crucial to warfare and before the invention of the steam engine, it was the fastest and most reliable form of land transport”* [5]. Despite the pivotal role horses have played in the development of human societies, the origin and history of modern breeds remain unclear in several aspects. For example, it is sometimes difficult to set apart the remains of wild and domestic horses. Contrary to dogs or pigs, the plasticity of the skeleton of the genus *Equus* is almost nil. As a result, even species as different as zebras and asses are almost indistinguishable based on their bones. Structural stability explains the problems that paleontologists encounter when attempting to work out the evolutionary history of horses [6]. To complicate matters further, the domestication of the horse did not result in any relevant changes in body size, as was the case with the remaining domesticates. This lack of diagnostic anatomical and biometrical criteria lies at the root of assigning status to archaeozoological remains [5]. It was for this reason that faunal analysts welcomed the introduction of molecular analyses some years ago.

Due to the high copy number of mtDNA in cells, their comparatively fast molecular evolution and their strictly maternal inheritance, over the last two decades mtDNA has become a valuable tool for phylogenetic and phylogeographic studies using ancient DNA analyses. However, both the lack of ancient sequences from pre-domestic and early domestic horses as well as the absence of a phylogeographic structure in modern breeds have prevented the establishment of any firm conclusions thus far. Previous studies have demonstrated the difficulty of restricting mitochondrial lineages to a particular geographic area or specific breed. To further complicate matters, the wild ancestor of domestic horses seems to be extinct since the agriotypic status of the few surviving Przewalski's horses is under debate [7]. Moreover, and in contrast with many other domesticated species [8]–[11], whereas an impressive amount of variability was discovered in modern horses in the mitochondrial control region [12]–[15], the species exhibits extremely low nucleotide diversity on the Y-chromosome. Recently the first description of two different Y- chromosomal haplotypes in Chinese indigenous horse breeds was reported [16]. Consequently, a sex bias has been assumed for the founders of domestic horses. It is likely that only a few stallions but many females were domesticated [17].

Even though several recent studies focused on the mtDNA variability of horses from restricted geographic regions and time periods [15], [18], some important issues remain to be solved, e.g. whether the large mtDNA variability was also present in the pre-domestic population and how this variation was distributed geographically. In this study, we attempt to shed new light on these issues by combining a large set of pre-domestic, early domestic and modern horse mtDNA sequences.

## Results

### Haplogroups and haplotypes

**Figure 1:**
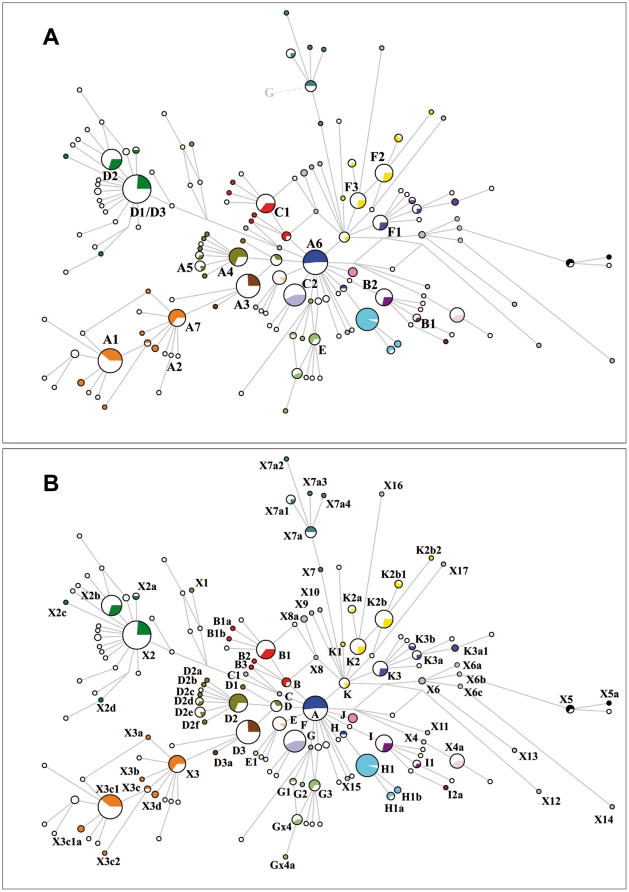
Median-joining network based on 247 bp of the mitochondrial D-Loop from 207 ancient samples (colored) and 601 modern sequences of primitive breeds (white). Eighty-seven haplotypes were found among the ancient samples; 103 different haplotypes among the primitive breeds and 36 haplotypes are shared (Table S5). Figure 1A shows haplotypes named after [13]–[14], whereas Figure 1B shows the new nomenclature (Table S4). Haplogroups are signed by a color code.

We successfully sequenced DNA from 85 out of 157 ancient samples resulting in a success rate of 54% (Table S1), i.e. 6 North East Siberian, 6 East Asian, 24 West and South Siberian, 6 Armenian, and 22 European samples (Iberia excluded), as well as 2 samples from Asia Minor and 19 Iberian remains. All sequences were deposited in Genbank (see Table S2 for accession numbers). In an attempt to improve the genetic information and the resolution of horse phylogeography we amplified the complete control region (D-loop) of the mitochondrial DNA (722 bp from 15,468–16,190 nps). But in contrast to other domestic species [19], we found that sequence length has no influence on phylogenetic resolution in horses and we decided to focus our analyses on the HVR1 region including the majority of Genbank sequences (247 bp from 15,494–15,740 nps). After their incorporation in our data set (85 sequences) of 122 previously published ancient sequences, the alignment of the 207 ancient sequences produced 78 variable sites (4 of which are indels) defining 113 haplotypes. Using all sequences (1754 modern horses and 207 ancient samples), we identified 4 strong mutational hotspots, 3 which were described in previous studies [13], [18] at nucleotide positions 15585, 15597 and 15650 and a new hotspot at position 15604. These positions were not included in the phylogenetic calculations. In addition, a few more mutational hotspots were individually down-weighted (Table S3). Once these mutational hotspots were eliminated from the dataset, the number of haplotypes fell to 87. Therefore, we introduced a new nomenclature for the haplotypes/groups (Figure 1 A, B; Table S4).

Strikingly, the inclusion of all the sequences available for modern horses (n = 1754) and ancient samples (n = 207) led to an unstructured and unresolved network. For this reason, the network (Figure 1B) was calculated focusing on 599 sequences from 46 primitive breeds, two Przewalski haplotypes and the 207 ancient samples. We divided the sequences into 19 haplogroups from A to K and from X1 to X7 based on the structure of the network (Figure 1B).

### Geographical origin of the mitochondrial lineages

**Figure 2:**
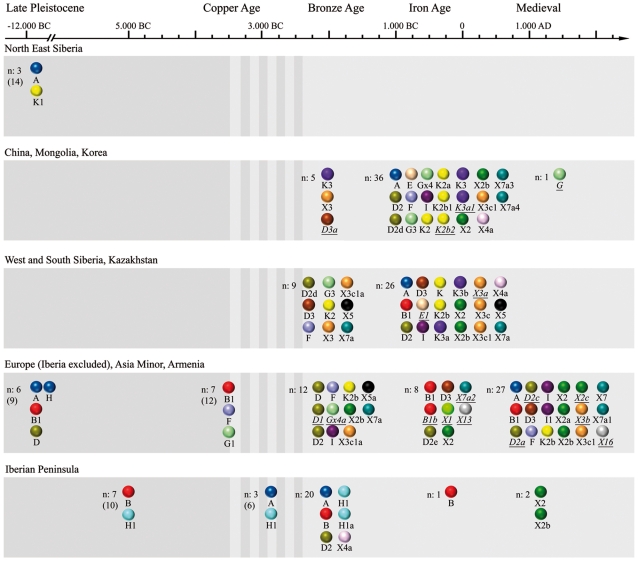
Origin of mitochondrial lineages of modern horses. Only mitochondrial lineages are shown which were/are present in domestic horses. Haplotypes in italic/underlined were so far only found in early-domestic remains. The striped zone indicates the postulated period of horse domestication.

Table 1 and Figure 2 give an overview of the geographic origin of mitochondrial lineages.

**Table 1:** Haplotype distribution.

			*LATE PLEISTOCENE*			*COPPER AGE*		*BRONZE AGE*				*IRON AGE*					*MEDIEVAL*		
		*n*	Alaska	North East Siberia	Europe	Europe Asia MinorARM	Iberia	China	West Siberia	Europe ARM	Iberia	North East Siberia	China	West South Siberia KZ	Europe	Iberia	Korea	Iberia	Europe (no Iberia)
***LATE PLEISTOCENE***	Alaska	***8***	**B2, X6, X6a–c, X14**																
	North East Siberia	***14***	**X8a**	**B3, C, G2, K1, X3d, X8, X10–12, X15**															
	Europe	***9***		**A**	**B1a, C1, X3c2, H**														
*COPPER AGE*	Europe Asia Minor Armenia	***12***			**B1**	**D2b, D2f, G1, X4, X9, X17**													
	Iberia	***13***					**B, H1, J**												
*BRONZE AGE*	China	***5***						**K3, D3a**											
	West Siberia	***9***				**F**		**X3**	**X5**										
	Europe/ARM	***12***			**D**	**F**			**X7a**	**D, D1, X3c1a, X5a**									
	Iberia	***23***		**A**			**B, H1, J**			**D2**	**B, H1, H1a**								
*IRONAGE*	North East Siberia	***1***										**X2d**							
	China	***36***		**A**		**F**		**K3**	**D2d, G3, K2, X3c1**	**D2, Gx4a, I, X2b**	**X4a**		**E, K2a, K2b1, K2b2, K3a1, X7a3, X7a4**						
	West South Siberia, Kazakhstan	***26***		**A**	**B1**				**D3, X3c1, X5, X7a**	**D2, I, K2b, X2b**	**X4a**		**X2**	**E1, K, K3a, K3b, X3a, X3c**					
	Europe	***8***			**B1**				**D3**				**X2**		**B1b, D2e, X1, X7a2, X13**				
	Iberia	***1***					**B**									**B**			
*MEDIEVAL*	Korea	***1***															**G**		
	Iberia	***2***								**X2b**			**X2**					**X2, X2b**	
	Europe (no Iberia)	***27***		**A**	**B1**	**F**			**D3, X3c1**	**I, K2b, X2b**			**X2**						**D2a, D2c, I1, X2a, X2c, X3b, X7, X7a1, X16**

Diagonal elements: private haplotypes; below diagonal: shared haplotypes. Grey-color code shows the frequency of shared haplotypes.

#### North and East Siberia

Only 2 out of the 12 Late Pleistocene lineages identified from 14 individuals are still present in modern breeds. Among these, basal haplotype A was recorded from the Late Pleistocene up to Mediaeval Times in several regions of Eurasia whereas K1 was only found in modern types. Only X8a was found in both Alaska and Siberia in the Late Pleistocene, although X8a is extinct today. X2d was only found in Iron Age wild horses but never in domestic horses, but haplogroup X2 is one of the most common haplogroups in modern breeds.

#### East Asia (China, Mongolia, and Korea)

No pre-domestic horse samples were available for this area. Haplotype D3a was found five times in Bronze Age samples. Haplotype K3 first occurred in China in the Bronze Age. Seven out of the 20 East Asian Iron Age lineages (n = 36) were only observed in this region, whereas 13 lineages were also present in other regions and times.

#### West and South Siberia, Kazakhstan

As was the case for East Asia, no data were available from pre-domestication times in this region. X3 appeared first in West Siberia during the Copper Age and, later, in China during the Bronze Age. X5 was confined to this region. The other eight Bronze Age lineages were also found in other regions. Six out of 18 Iron Age lineages (n = 26) were found only in this region.

#### Europe and Asia Minor (westwards from the Ural Mountains and the Caspian Sea to the Pyrenees, including Armenia)

We profited from the larger number of samples available for this region dating from the Late Pleistocene to Mediaeval Times. Interestingly, we found haplotype B1 before and after domestication in this region. Moreover, we found that 4 out of 7 lineages which were present among the Late Pleistocene horses (n = 9 samples), 6 out of 8 pre-domestic lineages (n = 12), 4 out of 11 Bronze Age lineages (n = 12), 5 out of 8 Iron Age lineages (n = 8) and 9 out of 18 Mediaeval lineages (n = 27) were confined to this region. Furthermore, 3 Late Pleistocene lineages, 2 pre-domestic lineages, 8 Bronze Age lineages, 3 Iron Age lineages, and 9 Mediaeval lineages were also found in other regions and times.

#### Iberian Peninsula

Three pre-domestic lineages (B, H1, J) (n = 13) were confined to this region. They were present during the Copper Age and Early Bronze Age. Haplotype B was also found in Iron Age horses. The dominating haplogroup during the Copper Age and Bronze Age was H1. However, the three Copper/Bronze Age haplotypes A, D2 and X4a were also found in other regions. Likewise, both Mediaeval lineages (X2, X2b) were found throughout Eurasia, probably due to the exchange of domestic animals.

#### Primitive horse breeds (Table S5)

**Figure 3:**
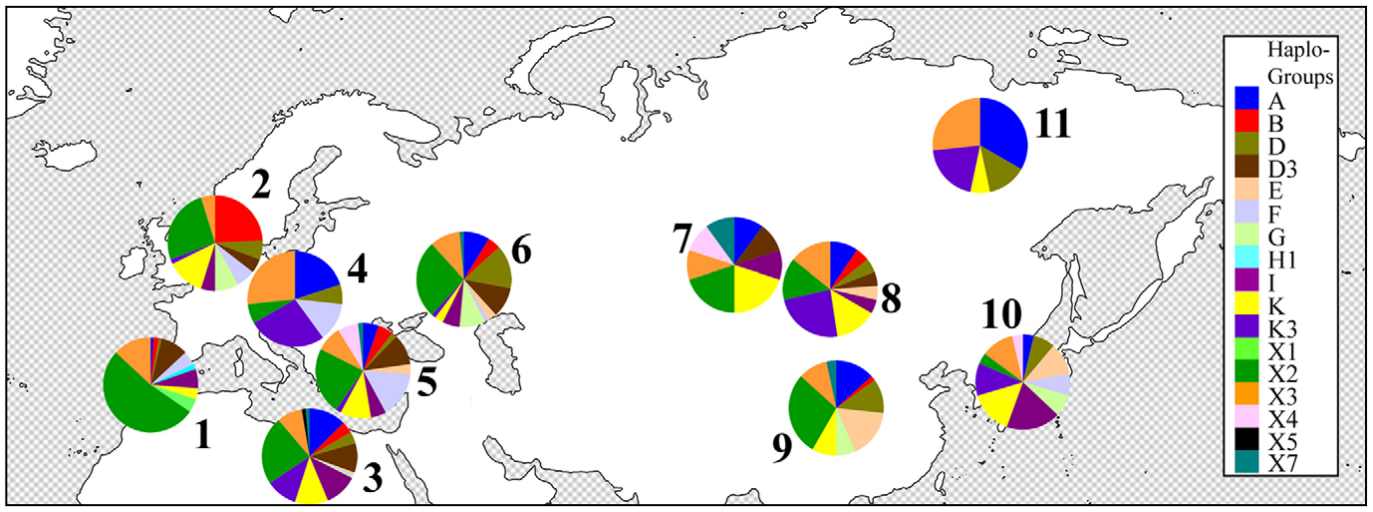
Haplogroup distribution of Eurasian primitive breeds (n = 601 animals; Table S13). (1: Asturcon (n = 12), Cartujano (n = 8), Garrano (n = 5), Losino (n = 10), Lusitano (n = 10), Marismeno (n = 12), Merens (n = 10), Pottoka (n = 13), Sorraia (n = 27); 2: Duelmener (n = 10), Exmoor (n = 17), Gotland (n = 3), Icelandic (n = 6), Norwegian Fjord (n = 11), Shetland (n = 18), Welsh (n = 1); 3: Arabian (n = 99), Barb (n = 37), Fulani (n = 9), Egyptian (n = 7); 4: Hucul (n = 11), Koonik (n = 5); 5: Anatolian (n = 17), Giara (n = 2), Mallorquina (n = 2), Pindos (n = 7), Sanfrantellano (n = 10), Sicilian Oriental (n = 1), Sicilian Ind. (n = 13), Skyros (n = 5); 6: Akhal Teke (n = 37), Caspian (n = 13), Vyatskaya (n = 18); 7: Tuva (n = 10); 8: Mongolian (n = 20), Przewalski (n = 2); 9: Debao (n = 24), Guan Mountains (n = 10), Guanzhong (n = 2), Wenshan (n = 2), XiNeHe (n = 1), Dali (n = 6), Yunnan (n = 1), Tibetan (n = 16); 10: Cheju (n = 25), Taishu (n = 2); 11: Yakut (n = 15)).

The haplogroup distribution of primitive breeds in Eurasia is shown in Figure 3. We found 39 out of 87 ancient lineages in our sample set of modern breeds (Table S6). Eleven out of these 39 haplotypes were lineages that were confined to a single primitive breed (B/Arabian; D2d/Cheju; G1/Akhal Teke; H/Garrano; H1/Marismeno; H1a/Lusitano; K2b1/Sicilian Oriental Purebred; K3b/ Yakut; X1/Pottoka; X2a/Debao; X3c/Lusitano; X5/Fulani). The haplotypes D, D2, E, K2 and X7a are rare or non-existent in Europe and Asia Minor; however, they are rather common in mainland Asia (Figure 3). Similarly, the A haplotype was not present in North West Europe or on the Iberian Peninsula. It is conspicuous that the K3 haplogroup is mainly present in Mongolia, Yakutia, Korea, Africa and Hungary. In contrast, haplotypes D3 and F are mainly present in Europe/Asia Minor, whereas B1 is particularly common in the Nordic region of Europe, and haplotype I appears especially on the Iberian Peninsula, Africa and Central Asia. K2b, X2, X2b, X3 and X3c1 are frequent and common haplotypes, and are more or less equally distributed across Eurasia.

### Presence of ancient haplotypes in modern horses

Thirty-nine of the 87 ancient haplotypes were also found in modern horses (Table S5). Today, X2 is the most common ancient haplotype in modern horses. X2 was found 326 times out of 1754 modern horse sequences. The second most common haplotype D3 was found 133 times followed by X2b (103), X3c1 (98), I (85), F (80), B1 (79), and A (75). The following 48 ancient lineages became extinct: B1a, *B1b*, B2, B3, C, C1, *D1*, *D2a*, D2b, *D2c*, D2f, *D3a*, *E1*, *G*, G2, *Gx4a*, H1b, I2a, J, *K2b2*, *K3a1*, *X1*, X2d, *X2c*, *X3a*, *X3b*, X3c1a, X3c2, X3d, X4, X5a, X6, X6a, X6b, X6c, X7, *X7a2*, X8, X8a, X9–12, *X13*, X14–15, *X16*, X17; italicized haplotypes were present in early domestic samples, but are extinct in modern horses today.

### Demography

**Figure 4:**
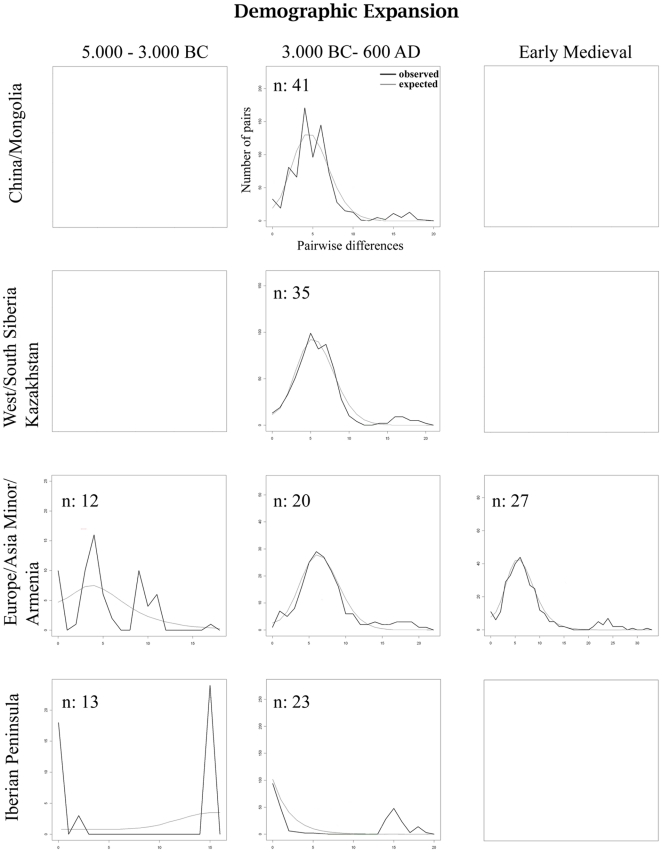
Mismatch distribution of mitochondrial haplotypes indicating population expansion (domestication) for all regions excluding Iberia during 3000 BC–600 AD.

The mismatch distributions of the West/South Siberian and European sample sets from early-domestic times show clear unimodal curves (Figure 4, Table S7). In contrast, the curves of the West/South Siberian sample sets were marked by a weak bimodal peak and the East Asian sample set from early domestic times has a ragged, bimodal or trimodal character. Finally, the curves of the Iberian pre-domestic and Copper/Bronze Age population, and the European samples of pre-domestic times were extremely ragged.

### Genetic variability

**Figure 5:**
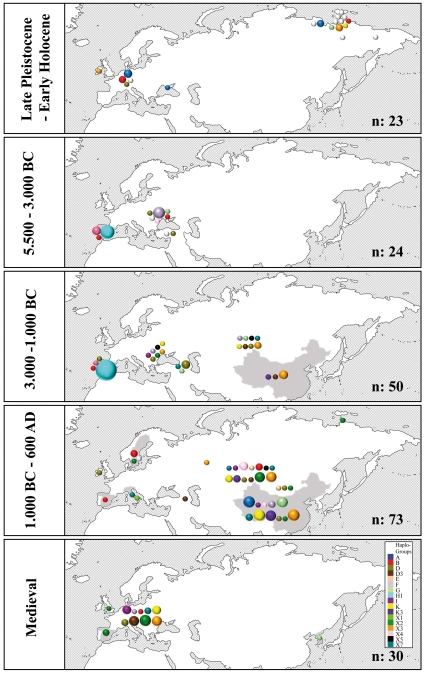
This figure illustrates the chronology of geographic haplogroup distribution. Size of circles represents haplogroup frequencies.

Nucleotide diversity varied between 0.034 (Europe 1000 BC–600 AD) and 0.005 (Iberia 5500–3000 BC), whereas haplotype diversity varied between 1.00 (West Siberia 3000–1000 BC; Europe 1000 BC–600 AD) and 0.60 (Iberia 5500–3000 BC). Altogether, the Iberian sets had the lowest values of both nucleotide and haplotype diversity (Table 2). The average nucleotide diversity of wild horses (Late Pleistocene and pre-domestic samples) was about 25% lower than the average of the domestic horses (Iberia was not included; for details see Table 2). A sub-division in the following three pre-domestic sub-populations (Table S8, Table S9) was supported by their Fst values ranging between 0.4 and 0.1: Alaskan horses, Iberian horses and Eurasian steppe horses. Nevertheless, some haplotypes were shared, indicating a common ancestry from a panmictic Late Pleistocene population or at least substantial gene flow between the regions. Furthermore, Fst values and haplotype composition did not support differentiation between horses from the Late Pleistocene and the Copper Age in Europe, as recently described based on shifts in their coat color [20], although, due to a lack of samples, this issue could not be addressed further. However, some lineages (e.g. E, E1) appeared later during the Bronze Age and Iron Age; others increased in frequency (e.g. X2, X3, D3), and still others became extinct soon after the glaciations (e.g. C, J, X8) (Figure 5).

**Table 2:** Nucleotide- and haplotype-diversity.

		*n*	No. of haplotpyes	Haplotype diversity	No. of poly-morphic sites	Nucleotide diversity
***LATE PLEISTOCENE***	*Alaska*	*8*	7	0.964+/−0.077	13	0.018+/−0.011
	*North East Siberia*	*14*	12	0.978+/−0.035	17	0.017+/−0.010
	*Europe*	*9*	7	0.917+/−0.092	11	0.013+/−0.008
***COPPER AGE***	*Europe/ Asia Minor Armenia*	*12*	8	0.849+/−0.104	18	0.016+/−0.010
	*Iberia*	*10*	3	0.600+/−0.131	4	0.005+/−0.004
***BRONZE AGE***	*China*	*5*	3	0.700+/−0.218	7	0.014+/−0.010
	*West Siberia*	*9*	9	1.000+/−0.052	17	0.021+/−0.013
	*Europe Armenia*	*12*	11	0.985+/−0.040	27	0.023+/−0.014
	*Iberia*	*38*	18	0.819+/−0.062	35	0.012+/−0.007
***IRON AGE***	*China*	*35*	20	0.952+/−0.019	31	0.021+/−0.011
	*West South Siberia, Kazakhstan*	*26*	18	0.969+/−0.018	30	0.025+/−0.014
	*Europe*	*8*	8	1.000+/−0.063	22	0.034+/−0.020
***MEDIEVAL***	*Europe*	*26*	19	0.966+/−0.022	32	0.027+/−0.015

## Discussion

A recent study pinpointed horse domestication in North Kazakhstan to around 3500 BC [21]. As demonstrated by our data, a large amount of genetic diversity was already present in pre-domestic horses before that time. Under normal circumstances, the bottleneck caused by domestication should have significantly reduced that genetic variation. As indicated by our data and previous studies [12], [13], [20], this has only partly been the case for horses. Altogether, 87 haplotypes were found in the archaeological remains (Pleistocene to Mediaeval Times). Overall, 32% (i.e., 9 out of 28 haplotypes) of the pre-domestic lineages (Pleistocene to Copper Age) have survived in the gene pool of domestic horses up to now. Seven of these pre-domestic lineages were also found in domestic horses from the Bronze Age and Iron Age and the remaining two were recorded in samples from the Mediaeval Ages. Considering all 87 of the ancient haplotypes, 29 haplotypes were found in Bronze Age samples, 47 haplotypes in Iron Age remains, and 21 haplotypes in Mediaeval bones. Our data provide evidence for multiple introgressions or, alternatively, for a second wave of domestication during the Iron Age, given that new 22 haplotypes were discovered in the gene pool of domestic horses for this period. In addition, ten haplotypes have their appearance during Mediaeval Times. However, we would like to raise a note of caution regarding the fact that, given the huge variation of mt DNA in horses, more ancient samples are necessary to determine the first occurrence of haplotypes in space and time. As is the case for all ancient DNA studies, we have a sample bias favoring modern sequences. Public databases contain 274 archived haplotypes of modern horses (19^th^ July 2010). Thus far, apparently only 45% of the mtDNA variation found in our study has survived in the modern domestic horses (i.e., 39 out of 87 haplotypes), and in terms of domestic samples between the Bronze Age and today, 30% (17 out of 56) of the maternal lineages have been lost during 5,500 years of horse breeding. This is still a large amount of genetic variation for a domestic animal. The reasons for the presence of such a large amount of genetic variation in the modern domestic horse may include one or a combination of the following: i) multiple origins; ii) an extremely large number of female founders and iii) large-scale introgression of local lineages into the domestic stock. In our opinion, it is not unlikely that all three of these reasons contributed evenly to dictate the present day state of affairs. Taken together, one major outcome of this study is that the huge diversity of the horse's mtDNA is not a consequence of animal breeding but instead a feature that was already present in the wild horse populations.

Our dataset has a bias of pre-domestic samples from Asia. For this reason one has to be careful when proposing putative centers of domestication. Notwithstanding, our results postulating local introgressions of wild mares and multiple domestication episodes in Europe and East Asia are not speculative. Both of these regions contributed significantly to determine the genetic portfolio of modern breeds during the Bronze and Iron Ages. Likewise, the mismatch distribution of the early-domestic European and Siberian horses produced evidence for a bottleneck 1500 years after the postulated origin of domestic horses in North Kazakhstan around 3500 BC [21]. Furthermore, our data on Bronze and Iron Age horses indicate local domestication and/or introgression of East Asian haplotypes. The evolution of these private East Asian haplotypes was enhanced by the isolation of Mongolian and Chinese wild horses due to the Altai-Mountains and the Takla Makan and Gobi deserts. These geographical barriers effectively segregated them from their cousins in the Eurasian steppe.

Our data have produced evidence for a panmictic horse population ranging from the Siberian Lowlands to the Pyrenees during the Late Pleistocene. The widespread distribution of haplogroups B1 (Europe, North East Siberia and Alaska), X8 (North East Siberia and Alaska), and X3 (North East Siberia and Ireland) as well as that of haplotype A (Germany, Ukraine and North East Siberia) lends support to the idea of widespread gene-flow across Eurasia. Later, in the Early Holocene and during the Copper Age, we find evidence for a genetic sub-division of Iberian and Eurasian steppe horses.

In Europe, we found three pre-domestic lineages (out of 8) that have survived in modern horses (Table S6). Two of them, B1 are still frequently present in modern European breeds, whereas G1 is now rare. In contrast, haplotype E and haplogroup K3 had their roots in East Asia, and both are common in East Asian primitive breeds today.

Separated from the rest of Europe by the Pyrenees, the history of the Iberian horses has to be considered independently. The Pyrenees effectively cut off the Iberian Peninsula from the rest of Eurasia and turned it into a glacial refuge. Pre-domestic to Copper Age Iberian horses were revealed as geographically isolated by our data. Interestingly, the haplotypes of the H1-group that dominated the ancient Iberian lineages during pre-domestic and early-domestic times are still occasionally present in Iberian breeds (e.g. Marismeño, Lusitano, Caballo de Corro) and also in the South American breeds (Argentinean Creole, Puerto Rican Paso Fino) today [18]. Remarkably, the ancient and singular Iberian haplotype B is nowadays found in Percheron (France), Arabian and Wielkopolski (Poland) horses. Such presence in the Percheron horses could be an indication of trade between Iberian and French people in ancient times, whereas the presence in the Arabian breed is likely a relic from the Moorish occupation of South Iberia. The Wielkopolski horse is a breed that also contains Arabian genes. We therefore agree with previous studies [18], [20] about the lack of evidence for an independent horse domestication process in Iberia, at least not on a significant scale, and provide instead strong evidence for introgression of local mares. Nowadays, all of the primitive Iberian breeds are dominated by haplotypes of the X3- and X2-haplogroups.

Considering our data from the Bronze Age horses, it appears that domestic horses spread soon after their initial domestication. Horse-riding was probably the catalyst for such fast spreading, starting an unprecedented process of gene flow 5,500 years ago. After 5,500 years of horse husbandry, the world is populated by 58 million domestic horses [FAO Statistics Division 2010 for 2008; http://faostat.fao.org]. The precise number of breeds is impossible to determine, but ranges from 600 up to 1000 breeds. Some of them are primitive breeds known since ancient times (e.g. Akhal Teke, Arab, Barb); others were founded a few decades ago (e.g. Lewitzer, Nederlands Mini Paarden, Pony of the Americas, Rocky Mountain Horse). However, many of the so-called “modern breeds” have their roots at the end of the eighteenth century or later [22]. Although inbreeding is sometimes used in animal husbandry for breed improvement, breed formation is often an open process. Crosses between different breeds or introgression of foreign specimens as well as introgression of wild horses have been reported [23]. For these reasons, mitochondrial lineage diversity changed over time by breeding or hybridization and introgression. As a result, a breed does not necessarily have a straightforward history and only rarely is it genetically isolated from the rest of the remaining population. All these processes have left their footprints in the genetic pedigree of the domestic horses. In fact, no other domestic animal today is less structured phylogenetically and phylogeographically speaking, or has an equivalent amount of mtDNA variation.

## Materials and Methods

### Samples

Our samples range from Alaska/North East Siberia to the Iberian Peninsula and cover a temporal window from the Late Pleistocene to Mediaeval Times (Table S1; ^14^C data are listed in Table S10). We successfully analyzed a total of 85 ancient Eurasian samples (out of 157). The sequence dataset obtained from those samples that yielded ancient DNA was supplemented by 122 ancient DNA sequences archived in GenBank (Table S2). In addition, 1754 GenBank sequences were incorporated from modern horses including the Przewalski horse (Table S11). We divided our dataset based on the geographic origin of the samples into: i) North East Siberia; ii) East Asia (e.g. China, Mongolia and Korea); iii) West and South Siberia (West Siberian Plain and Kazakhstan); iv) Europe and Asia Minor (westwards from the Ural Mountains and the Caspian Sea to the Pyrenees and Armenia); and v) Iberian Peninsula (south of the Pyrenees). Additionally, we considered the following time (cultural) periods: i) Late Pleistocene/Early Holocene; ii) Copper Age; iii) Bronze Age; iv) Iron Age; v) Early Mediaeval. It should be stressed that the range of dates of the chrono-cultural periods occasionally differs for each of the geographical regions.

### Ancient DNA extraction, amplification and sequencing

DNA was extracted from 250–400 mg samples of bone or tooth. The surfaces of these tissues were removed by abrasion to minimize contamination. Samples were ground to powder with a freezer mill and incubated in 0.45 M EDTA (pH 8.0) and 0.25 mg/ml Proteinase K overnight under rotation at room temperature. Afterwards, the pellets were collected by centrifugation for 5 min at 4,000 rpm in a Universal 320 centrifuge (Hettich). DNA was purified from the supernatant using a silica-based method as previously described [24]–[25].

Two different overlapping sets of primer were designed (Table S12). Using these primers, 722 bp (15.468–16.190 nps; primer set 1) or 713 bp (15.468–16.181 nps; primer set 2) were respectively amplified in a two-step multiplex PCR [26]–[27]. Overlapping PCR products including primers varied in length between 178 bp and 108 bp. We used 4 µl of extract in each multiplex PCR. The initial PCR was performed in a 20 µl reaction volume containing 1× AmpliTaq Gold PCR buffer II (ABI), 4 mM MgCl_2_, 1 mg/ml Bovine Serum Albumin (BSA), 250 µM each of dATP, dCTP and dGTP, 500 µM of dUTP, 150 nM of each primer and 2 U of AmpliTaq Gold (ABI). PCR was performed by adding 1 U of heat-labile Uracil-DNA Glycosylase (USB) and an initial incubation step of 15 min at 37°C to control for carry-over contamination. We used 5 µl of the diluted (1/30) PCR products for the next step (total reaction volume 20 µl). Singleplex PCRs contained 1× AmpliTaq Gold PCR Buffer II, 4 mM MgCl_2_, 1 mg/ml bovine serum albumin (BSA), 250 µM each of dATP, dCTP and dGTP, 500 µM of dUTP, 1.5 µM of each primer and 0.5 U of AmpliTaq Gold DNA polymerase. In both cases, PCR was run under the following conditions: denaturation and Taq activation at 94°C for 9 min followed by 30 to 35 cycles consisting of denaturation at 95°C for 20 sec, annealing temperature depending on the primer set (set I: 58°C; set II: 47°C) for 30 sec, elongation at 72°C for 30 sec and final extension at 72°C for 4 min. Negative extraction controls and negative PCR controls were performed for each PCR. Amplification products were visualized on agarose gel. Fragments were sequenced on an ABI PRISM 3730 capillary sequencer using the BigDye Terminator v3.1 cycle sequencing kit (Applied Biosystems).

### Authentication

DNA sampling, extractions and pre-PCR preparations were carried out in laboratories dedicated to ancient DNA following the standard procedures to avoid contamination. Direct sequencing was performed at least three times for each fragment from two independent extractions to detect PCR errors and miscoding of ancient DNA lesions from authentic sequences [28]. Independent replications were carried out in different laboratories for 21 bone samples (Humboldt University Berlin, Max Planck Institute Leipzig).

### Phylogenetic reconstructions

Sequences were truncated to nucleotide positions 15,494–15,740 (part of the mitochondrial HVR I) to accommodate published short sequences. Ancient sequences of the control region were aligned using BioEdit 7.0.9 (http://www.mbio.ncsu.edu/BioEdit/bioedit.html) based on a reference sequence [29]. Haplotypes were determined on the basis of informative substitutions excluding mutational hotspots [13] which were detected in a median joining network containing 1754 modern horses and 207 ancient samples. NETWORK 4.5.1.0 (http://www.fluxus-engineering.com) was used for calculation of the median joining network. Calculations were performed under the following conditions: deletions or insertions were double weighted; default setting of Epsilon (0) was chosen; transition/transversion ratio (TI/TV = 6.5). Variable sites were down- or up-weighted according to the maximal number of mutations at each position (Table S3). In addition to the three mutational hotspots identified in previous studies [13], [18], we excluded a fourth mutational hotspot. Thus, we defined new haplotypes and named them following a new nomenclature. Ancient haplotypes are named according to the letter of their haplogroup supplemented by a number or a number and a letter depending on how far they are from the basal node (for example: haplotype D3 is separated from D by 1 mutation, and haplotype D3a by 2 mutations). X indicates questionable haplogroups (X1–X8) and haplotypes (X9–X17), respectively. The phylogenetic position of these haplotypes depends strongly on the defaults of network reconstruction.

### mtDNA diversity and population structure

The genetic variability among ancient sequences was quantified as both nucleotide diversity and haplotype diversity. In addition, the average number of pairwise differences within and between populations and the population pairwise Fst values among populations (based on the Tamura & Nei distance; 10,000 permutations) were computed using Arlequin (http://cmpg.unibe.ch/software/arlequin3/). Demographic expansions (mismatch distribution) for certain time points and cultural horizons were estimated using Arlequin (demographic expansion) and figured using an Arlequin integrated R script (http://www.r-project.org/). The nucleotide diversity and mismatch distribution calculations were performed under the following setting: gamma shape parameter (α = 0.57) estimated in PAUP 4.0 (Sinauer Associates) using maximum likelihood.

## Supporting Information

Table S1 Samples analyzed for this study. The samples marked by a Y (Typing) gave a complete genotype. The extraction and amplification of the samples (Ext/Amp) and the reproduction (Rep) were performed in two institutes by Melanie Pruvost (MP) and Michael Cieslak (MC) at the Humboldt University in Berlin and Sebastian Lippold (SL) and Melanie Pruvost (MP) at the Max Planck Institute in Leipzig. EBA = Early Bronze Age; MED = Medieval Times.
(DOC)

Table S2 Samples analyzed for this study and samples from the Genbank. The samples highlighted in grey are the extended samples from the Genbank. The table includes the sample name, accession number, location, date and also an assigned haplogroup/haplotype name.
(DOC)

Table S3 Mitochondrial DNA control region (HVRI; position: 15.494–15.740) and the nucleotide characteristic data from the sample set of table S2 with additionally 207 ancient samples (Table S11).
(DOC)

Table S4 Variable positions of the d-loop sequences of ancient haplotypes. Nps less 15000 are shown. D2e corresponds to mtDNA reference, X79547 [23]. Mutational hotspots (15585, 15597, 15604 and 15650) are not included, because these positions were excluded in phylogenetic reconstructions. Gaps were signed by “-“. Previous nomenclature [13]–[14] is given in brackets.
(DOC)

Table S5 Ancient haplotypes occurrence in primitive breeds.
(DOC)

Table S6 Occurrence of ancient haplotypes in modern horses based on their Genbank entries.
(DOC)

Table S7 Sum of squared deviation and raggedness index (mismatch distribution).
(DOC)

Table S8 Population pairwise Fst values. Above diagonal: Matrix of significant Fst P values (p = 0.05). Below diagonal: Population pairwise Fst values.
(DOC)

Table S9 Average number of pairwise differences between and within populations. Above diagonal: Average number of pairwise differences between populations (PiXY); (grey = significant; p = 0.05). Diagonal elements: Average number of pairwise differences within population (PiX). Below diagonal: Corrected average pairwise difference (PiXY−(PiX+PiY)/2).
(DOC)

Table S10 ^14^C Dates of samples analyzed in this study. For details of samples see table 1. The Pleistocene samples are not directly dated, but estimated from context should be around 20,000 years old. The remaining dates are either calibrated carbon dates or derived from archaeological context.
(DOC)

Table S11 GenBank Accession no of modern horses (Przewalski horses are bold).
(DOC)

Table S12 Primer sequences.
(DOC)

Table S13 Primitive horse breeds and the Przewalski horse.
(DOC)
