# Supplementary material for: Origin and History of Mitochondrial DNA Lineages in Domestic Horses
Source: PLoS One. 2010 Dec 20;5(12):e15311. doi: 10.1371/journal.pone.0015311 (PMC3004868; doi:10.1371/journal.pone.0015311)
Supplement: Table S1 — Samples analyzed for this study. The samples marked by a Y (Typing) gave a complete genotype. The extraction and amplification of the samples (Ext/Amp) and the reproduction (Rep) were performed in two institutes by Melanie Pruvost (MP) and Michael Cieslak (MC) at the Humboldt University in Berlin and Sebastian Lippold (SL) and Melanie Pruvost (MP) at the Max Planck Institute in Leipzig. EBA = Early Bronze Age; MED = Medieval Times. (DOC) [file pone.0015311.s001.doc]

| **North East Siberia** (n: 7) | | | | | | | | | |
| --- | --- | --- | --- | --- | --- | --- | --- | --- | --- |
|  | **Sample** | **Accession** | **Ext/Amp** | **Rep.** | **Det.** | **Excavation** | **Geographical location** | **Date** | **Typing** |
| Late Pleistocene | SP1181A | FJ204314 | MP/MP | SL | W(ild) | Maliy Lyakhovsky Isl. | North Siberia | Pleistocene | Y(es) |
|  | SP1181B | FJ204315 | MP/MP |  | W | Bol'shoy Lyakhovsky Isl. | North Siberia | Pleistocene | Y |
|  | SP1181C | FJ204316 | MP/MP |  | W | Bol'shoy Lyakhovsky Isl. | North Siberia | Pleistocene | Y |
|  | SP1181D | FJ204324 | MP/MP |  | W | Bol'shoy Lyakhovsky Isl. | North Siberia | Pleistocene | Y |
|  | SP1181E | FJ204317 | MP/MP |  | W | Oyagosskiy Yar, Kondrat'evo R., mouth | Siberia | Pleistocene | Y |
|  | SP1181F | FJ204318 | MP/MP |  | W | Kotel'niy Isl., Anisiy Cape | Siberia | Pleistocene | Y |
| EBA | BAR 002 |  | MP/MP |  | D(om) | Preobrazhenka | Siberia | 2000-2500BC |  |
|  |  |  |  |  |  |  |  |  |  |
| **China, Mongolia** (n: 7) | | | | | | | | | |
| Iron Age | Fen 1 | FJ204377 | CW/MP |  | D | Fengtai | China (Qinghai) | 905-800 BC | Y |
|  | Fen 2 | FJ204378 | CW/MP |  | D | Fengtai | China (Qinghai) | 1000-800 BC | Y |
|  | Fen 3 | FJ204379 | CW/MP |  | D | Fengtai | China (Qinghai) | 1000-800 BC | Y |
|  | Fen 4 |  | CW/MP |  | D | Fengtai | China (Qinghai) | 1000-800 BC |  |
|  | OKG 001 | FJ204344 | MP/MP |  | D | Olon-Kurin-Gol 10 | Siberia (Mongolia) | 400-300 BC | Y |
|  | OKG 002 | FJ204345 | MP/MP |  | D | Olon-Kurin-Gol 10 | Siberia (Mongolia) | 400-300 BC | Y |
|  | OKG 003 | FJ204346 | MP/MP | MC | D | Olon-Kurin-Gol 10 | Siberia (Mongolia) | 400-300 BC | Y |
|  |  |  |  |  |  |  |  |  |  |
| **West and South Siberia** (n: 30) | | | | | | | | | |
| Bronze Age | TAR 001 | FJ204320 | MP/MP |  | D | Tartas1 | West Siberia | 2000 BC | Y |
|  | TAR 002 | FJ204321 | MP/MP |  | D | Tartas1 | West Siberia | 2000 BC | Y |
|  | TAR 004 | FJ204322 | MP/MP |  | D | Tartas1 | West Siberia | 2000 BC | Y |
|  | TAR 005 | FJ204323 | MP/MP |  | D | Tartas1 | West Siberia | 2000 BC | Y |
|  | TAR 006 |  | MP/MP |  | D | Tartas1 | West Siberia | 2000 BC |  |
|  | TAR 007 | FJ204325 | MP/MP |  | D | Tartas1 | West Siberia | 2000 BC | Y |
|  | TAR 008 | FJ204326 | MP/MP |  | D | Tartas1 | West Siberia | 2000 BC | Y |
|  | TAR 009 |  | MP/MP |  | D | Tartas1 | West Siberia | 2000 BC |  |
|  | TAR 010 | FJ204327 | MP/MP |  | D | Tartas1 | West Siberia | 2000 BC | Y |
|  | TAR 011 | FJ204328 | MP/MP |  | D | Tartas1 | West Siberia | 2000 BC | Y |
|  | BER 001 | FJ204319 | MP/MP |  | D | Denisova-Pescera | Siberia (Altai) | 3000 BC | Y |

| Iron Age | BER 002 | FJ204329 | MP/MP |  | D | Om-1 | Siberia (Altai) | 900BC | Y |
| --- | --- | --- | --- | --- | --- | --- | --- | --- | --- |
|  | Arz 1-2 |  | CW/MP |  | D | Arzan1 | South Siberia (Tuva) | 800BC |  |
|  | Arz 1-3 |  | CW/MP |  | D | Arzan1 | South Siberia (Tuva) | 800BC |  |
|  | Arz 2-1 | FJ204330 | CW/MP | MP | D | Arzan2 | South Siberia (Tuva) | 619-608 BC | Y |
|  | Arz 2-2 | FJ204331 | CW/MP | MP | D | Arzan2 | South Siberia (Tuva) | 619-608 BC | Y |
|  | Arz 2-3 | FJ204332 | CW/MP | MP | D | Arzan2 | South Siberia (Tuva) | 619-608 BC | Y |
|  | Arz 2-4 | FJ204333 | CW/MP | MP | D | Arzan2 | South Siberia (Tuva) | 619-608 BC | Y |
|  | Arz 2-5 | FJ204334 | CW/MP | MP | D | Arzan2 | South Siberia (Tuva) | 619-608 BC | Y |
|  | Arz 2-6 | FJ204335 | CW/MP | MP | D | Arzan2 | South Siberia (Tuva) | 619-608 BC | Y |
|  | Arz 2-7 | FJ204336 | CW/MP | MP | D | Arzan2 | South Siberia (Tuva) | 619-608 BC | Y |
|  | Arz 2-8 | FJ204337 | CW/MP | MP | D | Arzan2 | South Siberia (Tuva) | 619-608 BC | Y |
|  | Arz 2-9 | FJ204338 | CW/MP | MP | D | Arzan2 | South Siberia (Tuva) | 619-608 BC | Y |
|  | Arz 2-10 | FJ204339 | CW/MP | MP | D | Arzan2 | South Siberia (Tuva) | 619-608 BC | Y |
|  | Arz 2-11 | FJ204340 | CW/MP | MP | D | Arzan2 | South Siberia (Tuva) | 619-608 BC | Y |
|  | Arz 2-12 | FJ204341 | CW/MP | MP | D | Arzan2 | South Siberia (Tuva) | 619-608 BC | Y |
|  | Arz 2-13 | FJ204342 | CW/MP | MP | D | Arzan2 | South Siberia (Tuva) | 619-608 BC | Y |
|  | Arz 2-14 | FJ204343 | CW/MP | MP | D | Arzan2 | South Siberia (Tuva) | 619-608 BC | Y |
|  | Bars1A |  | CW/MP |  | D | Barsucij Log | South Siberia (Tuva) | 370-150 BC |  |
|  | Bars1B |  | CW/MP |  | D | Barsucij Log | South Siberia (Tuva) | 400-200 BC |  |
|  |  |  |  |  |  |  |  |  |  |
| **Europe, Asia Minor, Armenia** (n: 68; Spain not included) | | | | | | | | | |
| Late Glaciation-Mesolithic | PET1 | FJ204352 | SL/SL |  | W | Petersfels | South Germany | 14000-11000 BC | Y |
|  | PET2 |  | SL/SL |  | W | Petersfels | South Germany | 14000-11000 BC |  |
|  | PET3 |  | SL/SL |  | W | Petersfels | South Germany | 14000-11000 BC |  |
|  | PET5 |  | SL/SL |  | W | Petersfels | South Germany | 14000-11000 BC |  |
|  | PET6 |  | SL/SL |  | W | Petersfels | South Germany | 14000-11000 BC |  |
|  | Kg1 | FJ204347 | SL/SL |  | W | Kniegrotte | Germany (Thuringia) | 15000-14000 BC | Y |
|  | Kg2 | FJ204348 | SL/SL | MP | W | Kniegrotte | Germany (Thuringia) | 15000-14000 BC | Y |
|  | Kg3 |  | SL/SL |  | W | Kniegrotte | Germany (Thuringia) | 15000-14000 BC |  |
|  | Kg4 |  | SL/SL |  | W | Kniegrotte | Germany (Thuringia) | 15000-14000 BC |  |
|  | Kg5 | FJ204351 | SL/SL |  | W | Kniegrotte | Germany (Thuringia) | 15000-14000 BC | Y |
|  | Spa 1 | FJ204354 | CW/MP |  | W | Span-Koba | Ukraine (Peninsula Crimea) | 9390-9210 BC | Y |
| Eneolithic | TRE1 |  | MP/MP |  | W | Trestiana | Romania | 5700-5600 BC |  |
|  | TRE2 |  | MP/MP |  | W | Trestiana | Romania | 5700-5600 BC |  |
|  | TRE3 |  | MP/MP |  | W | Trestiana | Romania | 5700-5600 BC |  |
|  | BUP1 |  | MP/MP |  | W | Bucsani Pod | Romania | 5500-5000 BC |  |
|  | ISA1 |  | MP/MP |  | W | Isaia | Romania | 5500 BC |  |
|  | ISA2 |  | MP/MP |  | W | Isaia | Romania | 5500 BC |  |
|  | HAR1 |  | MP/MP |  | W | Harsova | Romania | 5000-5500 BC |  |
|  | HAR2 |  | MP/MP |  | W | Harsova | Romania | 4500-2000 BC |  |
|  | HAR3 |  | MP/MP |  | W | Harsova | Romania | 4500-2000 BC |  |
|  | Pie7 |  | MP/MP |  | W | Pietrele | Romania | 4300 BC |  |
|  | Pie9 | FJ204355 | MP/MP |  | W | Pietrele | Romania | 4300 BC | Y |
|  | Pie11 |  | MP/MP |  | W | Pietrele | Romania | 4300 BC |  |
|  | VIT1 |  | MP/MP |  | W | Vitanesti | Romania | 4300-4220 BC |  |
|  | VIT2 | FJ204357 | MP/MP |  | W | Vitanesti | Romania | 4350-4220 BC | Y |
|  | VIT3 |  | MP/MP |  | W | Vitanesti | Romania | 4300-4220 BC |  |
|  | VIT4 |  | MP/MP |  | W | Vitanesti | Romania | 4360-4220 BC |  |
|  | ORL1 |  | MP/MP |  | W | Orlovka | Moldova | 4000 BC |  |
|  | ORL2 |  | MP/MP |  | W | Orlovka | Moldova | 4000 BC |  |
|  | ORL3 |  | MP/MP |  | W | Orlovka | Moldova | 4000 BC |  |
|  | ORL4 | FJ204358 | MP/MP |  | W | Orlovka | Moldova | 4000 BC | Y |
| Copper Age | CAS1 | FJ204356 | MP/MP |  | W | Cascioarele | Romania | 3700-3380 BC | Y |
|  | MAY1 | FJ204359 | MP/MP |  | W | Mayaki | Ukraine | 3600-3100 BC | Y |
|  | MAY2 |  | MP/MP |  | W | Mayaki | Ukraine | 3600-3100 BC |  |
|  | MAY3 |  | MP/MP |  | W | Mayaki | Ukraine | 3640-3490 BC |  |
|  | MAY4 | FJ204364 | MP/MP |  | W | Mayaki | Ukraine | 3600-3100 BC | Y |
|  | MAY5 | FJ204360 | MP/MP |  | W | Mayaki | Ukraine | 3250-3100 BC | Y |
|  | MAY6 | FJ204361 | MP/MP |  | W | Mayaki | Ukraine | 3520-3330 BC | Y |
|  | MAY7 | FJ204362 | MP/MP |  | W | Mayaki | Ukraine | 3520-3380 BC | Y |
|  | MAY8 |  | MP/MP |  | W | Mayaki | Ukraine | 3600-3100 BC |  |
|  | MAY9 |  | MP/MP |  | W | Mayaki | Ukraine | 3600-3100 BC |  |
|  | MAY10 | FJ204363 | MP/MP |  | W | Mayaki | Ukraine | 3650-3500 BC | Y |
|  | MOL5 |  | MP/MP |  | W? | Molyukhov Bugor | Ukraine | 3720-3630 BC |  |
|  | MOL7 |  | MP/MP |  | W? | Molyukhov Bugor | Ukraine | 3720-3630 BC |  |
|  | MOL8 |  | MP/MP |  | W? | Molyukhov Bugor | Ukraine | 3720-3630 BC |  |
| EBA | Kan3 | FJ204353 | CW/CW |  | W? | Kirklareli-Kanligecit | Turkey | 3850 BC | Y |
|  | Kan5 | FJ204349 | CW/CW |  | W? | Kirklareli-Kanligecit | Turkey | 3850 BC | Y |
| Bronze Age | GRO8 |  | MP/MP |  | D | Großobringen | Germany | 3000-2500 BC |  |
|  | GRO9 |  | MP/MP |  | D | Großobringen | Germany | 3000-2500 BC |  |
|  | GRO10 |  | MP/MP |  | D | Großobringen | Germany | 3000-2500 BC |  |
|  | GRO11 |  | MP/MP |  | D | Großobringen | Germany | 3000-2500 BC |  |
|  | MOH1 | FJ204365 | CW/MP |  | D | Mohra-Blur | Armenia | 3000 BC | Y |
|  | Gar1 |  | MP/MP |  | D | Garbovat | Romania | 1500-1000 BC |  |
|  | Gar2 |  | MP/MP |  | D | Garbovat | Romania | 1500-1000 BC |  |
|  | Gar3 | FJ204366 | MP/MP |  | D | Garbovat | Romania | 1500-1000 BC | Y |
|  | Gar4 | FJ204367 | MP/MP |  | D | Garbovat | Romania | 1500-1000 BC | Y |
|  | Bar1 |  | MP/MP |  | D | Garbovat | Romania | 1500-1000 BC |  |
|  | Bar3 |  | MP/MP |  | D | Garbovat | Romania | 1500-1000 BC |  |
|  | Lch 1 | FJ204370 | CW/MP |  | D | Lchashen | Armenia | 1410-1250 BC | Y |
|  | Lor3 | FJ204350 | CW/MP |  | D | Lori-Berd | North Armenia | 1950-1750 BC | Y |
|  | Lor2 | FJ204368 | CW/MP |  | D | Lori-Berd | North Armenia | 1950-1750 BC | Y |
|  | Lor 1 | FJ204371 | CW/MP |  | D | Lori-Berd | North Armenia | 1950-1750 BC | Y |
|  | Mic1 | FJ204372 | MP/MP |  | D | Miciurin | Moldova | 1500-1000 BC | Y |
|  | Mic2 | FJ204373 | MP/MP |  | D | Miciurin | Moldova | 1500-1000 BC | Y |
|  | Mic3 | FJ204374 | MP/MP |  | D | Miciurin | Moldova | 1500-1000 BC | Y |
|  | Mic4 | FJ204375 | MP/MP |  | D | Miciurin | Moldova | 1500-1000 BC | Y |
|  | Mic5 | FJ204376 | MP/MP |  | D | Miciurin | Moldova | 1500-1000 BC | Y |
|  | Shi 1 | FJ204369 | CW/MP |  | D | Shirakavan | Armenia | 895-795 BC | Y |

| **Iberian Peninsula** (n: 45) | | | | | | | | | |
| --- | --- | --- | --- | --- | --- | --- | --- | --- | --- |
| Mesolithic-Neolithic | 41 |  | MC/MC |  | W | Atxoste | Spain | 5500-4950 BC |  |
|  | 42 |  | MC/MC |  | W | Atxoste | Spain | 5500-4950 BC |  |
|  | 43 |  | MC/MC |  | W | Atxoste | Spain | 5500-4950 BC |  |
|  | 44 | FJ204384 | MC/MC |  | W | Atxoste | Spain | 5500-4950 BC | Y |
|  | 45 | FJ204380 | MC/MC |  | W | Atxoste | Spain | 5500-4950 BC | Y |
|  | 1 | FJ204390 | MC/MC |  | W | Cueva Fosca -Valencia-Cartellon | Spain | 5200 -4900BC | Y |
|  | 2 | HM802276 | MC/MC |  | W | Cueva Fosca -Valencia-Cartellon | Spain | 5200 -4900BC | Y |
|  | 3 | FJ204381 | MC/MC |  | W | Cueva Fosca -Valencia-Cartellon | Spain | 5200-4900 BC | Y |
|  | 31 | FJ204382 | MC/MC |  | W | Cueva Fosca -Valencia-Cartellon | Spain | 5210-4910 BC | Y |
|  | 32 | FJ204383 | MC/MC |  | W | Cueva Fosca -Valencia-Cartellon | Spain | 5220-4980 BC | Y |
|  | 33 |  | MC/MC |  | W | Cueva Fosca -Valencia-Cartellon | Spain | 5220-4900 BC |  |
|  | 34 |  | MC/MC |  | W | Cueva Fosca -Valencia-Cartellon | Spain | 5220-4900 BC |  |
|  | 35 | HM802280 | MC/MC |  | W | Cueva Fosca -Valencia-Cartellon | Spain | 5380-5210BC | Y |
|  | 36 |  | MC/MC |  | W | Cueva Fosca -Valencia-Cartellon | Spain | 5070-4840BC |  |
|  | 37 | FJ204385 | MC/MC |  | W | Cueva Fosca -Valencia-Cartellon | Spain | 5210-4910 BC | Y |
|  | 17 | FJ204386 | MC/MC | SL | W | Cueva De La Vaquera-Segovia | Spain | 5210-4940 BC | Y |
| Copper Age | 27 | HM802281 | MC/MC |  | D | El Caprichio-Madrid | Spain | 4300-2200 BC | Y |
|  | 28 |  | MC/MC |  | D | Carmona-Sevillia/Andalusia | Spain | 4300-2200 BC |  |
|  | 19 |  | MC/MC |  | D | Las Pozas -Zamora | Spain | 4300 -2200 BC |  |
|  | 20 | HM802277 | MC/MC | SL | D | Cueva Rubia-Valmayor/Madrid | Spain | 2880-2570 BC | Y |
|  | 21 | HM802278 | MC/MC |  | D | Cueva Rubia-Valmayor/Madrid | Spain | 2900-2500 BC | Y |
| Bronze Age | 22 |  | MC/MC |  | D | Cueva Rubia-Valmayor/Madrid | Spain | 1350 BC |  |
|  | 23 | HM802279 | MC/MC |  | D | Cueva Rubia-Valmayor/Madrid | Spain | 1350 BC | Y |
|  | 24 | FJ204389 | MC/MC |  | D | Cueva Rubia-Valmayor/Madrid | Spain | 1350 BC | Y |
|  | 25 |  | MC/MC |  | D | Cueva Rubia-Valmayor/Madrid | Spain | 1350 BC |  |
|  | 26 |  | MC/MC |  | D | Cueva Rubia-Valmayor/Madrid | Spain | 1350 BC |  |
|  | 38 |  | MC/MC |  | D | El Acequion | Spain | 2200-800 BC |  |
|  | 39 | FJ204387 | MC/MC |  | D | El Acequion | Spain | 2200-800 BC | Y |
|  | 40 | FJ204388 | MC/MC |  | D | El Acequion | Spain | 2200-800 BC | Y |
|  | 8 |  | MC/MC |  | D | Peñalosa-Jaén -Andalucia | Spain | 2200 - 800 BC |  |
|  | 9 |  | MC/MC |  | D | Peñalosa-Jaén -Andalucia | Spain | 2200 - 800 BC |  |
|  | 10 |  | MC/MC |  | D | Peñalosa-Jaén -Andalucia | Spain | 2200 - 800 BC |  |
|  | 11 |  | MC/MC |  | D | Peñalosa-Jaén -Andalucia | Spain | 2200 - 800 BC |  |
|  | 12 |  | MC/MC |  | D | Peñalosa-Jaén -Andalucia | Spain | 2200 - 800 BC |  |
|  | 13 |  | MC/MC |  | D | Peñalosa-Jaén -Andalucia | Spain | 2200 - 800 BC |  |
|  | 14 |  | MC/MC |  | D | Peñalosa-Jaén -Andalucia | Spain | 2200 - 800 BC |  |
|  | 15 |  | MC/MC |  | D | Peñalosa-Jaén -Andalucia | Spain | 2200 - 800 BC |  |
|  | 18 |  | MC/MC |  | D | Morra Del Quintanar- Albacete | Spain | 2200 - 800 BC |  |
| Iron Age | 16 |  | MC/MC |  | D | La Mota -Medina Del Campo | Spain | 800 BC - 6 AD |  |
|  | 4 | FJ204391 | MC/MC | SL | W? | Soto de Medinilla -Valladolid | Spain | 800 BC - 6 AD | Y |
|  | 5 |  | MC/MC |  | W? | Soto de Medinilla -Valladolid | Spain | 800 BC - 6 AD |  |
|  | 6 |  | MC/MC |  | W? | Soto de Medinilla -Valladolid | Spain | 800 BC - 6 AD |  |
|  | 7 |  | MC/MC |  | W? | Soto de Medinilla -Valladolid | Spain | 800 BC - 6 AD |  |
| Med. | 29 | FJ204392 | MC/MC |  | D | Mucientes-Valladolid | Spain | 660-780 AD | Y |
|  | 30 |  | MC/MC |  | D | Mucientes-Valladolid | Spain | 680-890 AD |  |
