# Supplementary material for: Origin and History of Mitochondrial DNA Lineages in Domestic Horses
Source: PLoS One. 2010 Dec 20;5(12):e15311. doi: 10.1371/journal.pone.0015311 (PMC3004868; doi:10.1371/journal.pone.0015311)
Supplement: Table S2 — Samples analyzed for this study and samples from the Genbank. The samples highlighted in grey are the extended samples from the Genbank. The table includes the sample name, accession number, location, date and also an assigned haplogroup/haplotype name. (DOC) [file pone.0015311.s002.doc]

| **Alaska** | | | | | |
| --- | --- | --- | --- | --- | --- |
|  | **Haplotype** | **Sample** | **Accession** | **Geographical location** | **Date** |
| LATE PLEISTOCENE | B2 |  | AF326672 | Alaska | Late Pleistocene |
|  | X6a |  | AF326671 | Alaska | Late Pleistocene |
|  | X6b |  | AF326675 | Alaska | Late Pleistocene |
|  | X6 |  | AF326669 | Alaska | Late Pleistocene |
|  | X6 |  | AF326670 | Alaska | Late Pleistocene |
|  | X6c |  | AF326673 | Alaska | Late Pleistocene |
|  | X14 |  | AF326674 | Alaska | Late Pleistocene |
|  | X8a |  | AF326668 | Alaska | Late Pleistocene |
|  |  |  |  |  |  |
| **North East Siberia** | | | | | |
| LATE PLEISTOCENE | X8a | SP1181A | FJ204314 | Maliy Lyakhovsky Isl., N-E Siberia | Late Pleistocene |
|  | C | SP1181B | FJ204315 | Bol'shoy Lyakhovsky Isl., N-E Siberia | Late Pleistocene |
|  | X12 | SP1181C | FJ204316 | Bol'shoy Lyakhovsky Isl., N-E Siberia | Late Pleistocene |
|  | X3d | SP1181D | FJ204324 | Bol'shoy Lyakhovsky Isl., N-E Siberia | Late Pleistocene |
|  | G2 |  | DQ007582 | Yana R. Lower course, N-E Siberia | Late Pleistocene |
|  | K1 |  | DQ007575 | Bol. Lyakhovsky Island, N-E Siberia | 34800±1000 BP |
|  | A |  | DQ007580 | Lena R. Delta, N-E Siberia | Late Pleistocene |
|  | A |  | DQ007577 | Lena R. Delta, N-E Siberia | 31220±180 BP |
|  | B3 |  | DQ007574 | Bol. Lyakhovsky Island, N-E Siberia | Late Pleistocene |
|  | X10 |  | DQ007578 | Bykovsky Peninsula, Lena Delta, N-E Siberia | Late Pleistocene |
|  | X11 |  | DQ007576 | Ulakhan-Sullar, Adycha R., Yana Basin, N-E Siberia | Late Pleistocene |
|  | X15 |  | DQ007579 | Alyoshkina, Kolyma R., N-E Siberia | Late Pleistocene |
|  | X8 | SP1181E | FJ204317 | Oyagosskiy Yar, Kondrat'evo R., mouth, N-E Siberia | Late Pleistocene |
|  | X3d | SP1181F | FJ204318 | Kotel'niy Isl., Anisiy Cape, N-E Siberia | Late Pleistocene |
| IRON AGE | X2d |  | DQ007573 | Bol. Lyakhovsky Island, N-E Siberia | 2,220±50 BP |

| **China** |  |  |  |  |  |
| --- | --- | --- | --- | --- | --- |
| BRONZE AGE | X3 |  | DQ900925 | Dashanqian site, Inner Mongolia, China | 2000 BC |
|  | X3 |  | DQ900923 | Dashanqian site, Inner Mongolia, China | 2000 BC |
|  | X3 |  | DQ900922 | Dashanqian site, Inner Mongolia, China | 2000 BC |
|  | K3 |  | DQ900924 | Dashanqian site, Inner Mongolia, China | 2000 BC |
|  | D3a |  | DQ900926 | Dashanqian site, Inner Mongolia, China | 2000 BC |
| IRON AGE | G3 | Fen 1 | FJ204377 | China (Qinghai) | 905-800 BC |
|  | A | Fen 2 | FJ204378 | China (Qinghai) | 1000-800 BC |
|  | Gx4 | Fen 3 | FJ204379 | China (Qinghai) | 1000-800 BC |
|  | G3 |  | EU931592 | Xindianzi site, China | 500 BC |
|  | G3 |  | EU931595 | Xindianzi site, China | 500 BC |
|  | F |  | EU931598 | Banchneng site, China | 500 BC |
|  | F |  | EU931600 | Banchneng site, China | 500 BC |
|  | X4a |  | EU931597 | Banchneng site, China | 500 BC |
|  | I |  | EU931607 | Yujiazhuang site, China | 500 BC |
|  | A |  | EU931601 | Banchneng site, China | 500 BC |
|  | A |  | EU931609 | Maoyuan site, China | 500 BC |
|  | A |  | EU931604 | Xiaoshuanggucheng site, China | 500 BC |
|  | A |  | EU931594 | Xindianzi site, China | 500 BC |
|  | X7a4 |  | EU931605 | Xiaoshuanggucheng site, China | 500 BC |
|  | X7a3 |  | EU931591 | Xindianzi site, China | 500 BC |
|  | K2 |  | EU931608 | Yujiazhuang site, China | 500 BC |
|  | K2 |  | EU931606 | Xiaoshuanggucheng site, China | 500 BC |
|  | K2b2 |  | EU931588 | Xindianzi site, China | 500 BC |
|  | K2b1 |  | EU931593 | Xindianzi site, China | 500 BC |
|  | K3a1 |  | EU931584 | Xindianzi site, China | 500 BC |
|  | K3a1 |  | EU931586 | Xindianzi site, China | 500 BC |
|  | K3 |  | EU931602 | Banchneng site, China | 500 BC |
|  | K3 |  | EU931590 | Xindianzi site, China | 500 BC |
|  | D2d |  | EU931587 | Xindianzi site, China | 500 BC |
|  | X2 |  | EU931599 | Banchneng site, China | 500 BC |
|  | X3c1 |  | EU931596 | Banchneng site, China | 500 BC |
|  | X3c1 |  | EU931585 | Xindianzi site, China | 500 BC |
|  | X3c1 |  | EU931589 | Xindianzi site, China | 500 BC |
|  | X3c1 |  | EU931603 | Xiaoshuanggucheng site, China | 500 BC |
|  | X2b | OKG 001 | FJ204344 | Siberia (Mongolia) | 400-300 BC |
|  | D2 | OKG 002 | FJ204345 | Siberia (Mongolia) | 400-300 BC |
|  | E | OKG 003 | FJ204346 | Siberia (Mongolia) | 400-300 BC |
|  | X3c1 |  | DQ900928 | China Jinggouzi site, Inner Mongolia | 100 BC |
|  | Gx4 |  | DQ900927 | China Jinggouzi site, Inner Mongolia | 100 BC |
|  | K2a |  | DQ900930 | China Jinggouzi site, Inner Mongolia | 100 BC |
|  | K2b1 |  | DQ900929 | China Jinggouzi site, Inner Mongolia | 100 BC |

| **Korea** |  |  |  |  |  |
| --- | --- | --- | --- | --- | --- |
| MEDIEVAL | G |  | AY049720 | Korea | 700-800 AD |
|  |  |  |  |  |  |
| **West and South Siberia, Kazakhstan** | | | |  |  |
| EARLY BRONZE AGE | X5 | TAR 001 | FJ204320 | Tartas1 , West Siberia | 2000BC |
|  | D3 | TAR 002 | FJ204321 | Tartas1 , West Siberia | 2000BC |
|  | K2 | TAR 004 | FJ204322 | Tartas1 , West Siberia | 2000BC |
|  | G3 | TAR 005 | FJ204323 | Tartas1 , West Siberia | 2000BC |
|  | X3c1a | TAR 007 | FJ204325 | Tartas1 , West Siberia | 2000BC |
|  | X3 | TAR 008 | FJ204326 | Tartas1 , West Siberia | 2000BC |
|  | F | TAR 010 | FJ204327 | Tartas1 , West Siberia | 2000BC |
|  | X7a | TAR 011 | FJ204328 | Tartas1 , West Siberia | 2000BC |
|  | D2d | BER 001 | FJ204319 | Denisova-Pescera, West Siberia (Altai) | 3000 BC |
| IRON AGE | X2 | BER 002 | FJ204329 | Om-1, West Siberia (Altai) | 900 BC |
|  | X3c |  | DQ007571 | Russia/Ural | 213±34 BC |
|  | X4a |  | AJ876887 | Berel, Kazakhstan | 300 BC |
|  | B1 |  | AJ876889 | Berel, Kazakhstan | 300 BC |
|  | I |  | AJ876888 | Berel, Kazakhstan | 300 BC |
|  | K3b |  | AJ876891 | Berel, Kazakhstan | 300 BC |
|  | D2 |  | AJ876892 | Berel, Kazakhstan | 300 BC |
|  | D3 |  | AJ876890 | Berel, Kazakhstan | 300 BC |
|  | X2 |  | AJ876885 | Berel, Kazakhstan | 300 BC |
|  | X2 |  | AJ876884 | Berel, Kazakhstan | 300 BC |
|  | A |  | AJ876883 | Berel, Kazakhstan | 300 BC |
|  | A |  | AJ876886 | Berel, Kazakhstan | 300 BC |
|  | X2b | Arz 2-1 | FJ204330 | South Siberia (Tuva) | 619-608 BC |
|  | X3a | Arz 2-2 | FJ204331 | South Siberia (Tuva) | 619-608 BC |
|  | X2b | Arz 2-3 | FJ204332 | South Siberia (Tuva) | 619-608 BC |
|  | X3c1 | Arz 2-4 | FJ204333 | South Siberia (Tuva) | 619-608 BC |
|  | X3c1 | Arz 2-5 | FJ204334 | South Siberia (Tuva) | 619-608 BC |
|  | K3a | Arz 2-6 | FJ204335 | South Siberia (Tuva) | 619-608 BC |
|  | X4a | Arz 2-7 | FJ204336 | South Siberia (Tuva) | 619-608 BC |
|  | K | Arz 2-8 | FJ204337 | South Siberia (Tuva) | 619-608 BC |
|  | K2b | Arz 2-9 | FJ204338 | South Siberia (Tuva) | 619-608 BC |
|  | B1 | Arz 2-10 | FJ204339 | South Siberia (Tuva) | 619-608 BC |
|  | X5 | Arz 2-11 | FJ204340 | South Siberia (Tuva) | 619-608 BC |
|  | X7a | Arz 2-12 | FJ204341 | South Siberia (Tuva) | 619-608 BC |
|  | X4a | Arz 2-13 | FJ204342 | South Siberia (Tuva) | 619-608 BC |
|  | E1 | Arz 2-14 | FJ204343 | South Siberia (Tuva) | 619-608 BC |

| **Europe (Germany, Ireland, Ukraine)** | | | |  |  |
| --- | --- | --- | --- | --- | --- |
| LATE PLEISTOCENE-MESOLITHIC | B1a | PET1 | FJ204352 | Petersfels, South Germany | 14000-11000 BC |
|  | A | Kg1 | FJ204347 | Kniegrotte, Germany (Thuringia) | 15000-14000 BC |
|  | H | Kg2 | FJ204348 | Kniegrotte, Germany (Thuringia) | 15000-14000 BC |
|  | C1 |  | DQ007556 | Hohlefels, Germany | 12550±60 BP |
|  | D |  | DQ007558 | Petersfels, Germany | 12,545±50 BP |
|  | A |  | DQ007591 | Vogelherd IV, Germany | 13.845±50 BP |
|  | B1 | Kg5 | FJ204351 | Kniegrotte, Germany (Thuringia) | 15000-14000 BC |
|  | X3c2 |  | DQ327850 | Waterford, Shandon, Ireland | 25624+/-400 BC |
|  | A | Spa 1 | FJ204354 | Span-Koba, Ukraine (Peninsula Crimea) | 9390-9210 BC |
|  |  |  |  |  |  |
| **Europe, Asia Minor, Armenia** | | |  |  |  |
| ENEOLTIHIC | X4 | Pie9 | FJ204355 | Pietrele, Romania | 4300 BC |
|  | D2b | VIT2 | FJ204357 | Vitanesti, Romania | 4350-4220 BC |
|  | F | ORL4 | FJ204358 | Orlovka, Moldova | 4000 BC |
| COPPER AGE | G1 | CAS1 | FJ204356 | Cascioarele, Romania | 3700-3380 BC |
|  | F | MAY1 | FJ204359 | Mayaki, Ukraine | 3600-3100 BC |
|  | F | MAY4 | FJ204364 | Mayaki, Ukraine | 3600-3100 BC |
|  | B1 | MAY5 | FJ204360 | Mayaki, Ukraine | 3250-3100 BC |
|  | F | MAY6 | FJ204361 | Mayaki, Ukraine | 3520-3330 BC |
|  | F | MAY7 | FJ204362 | Mayaki, Ukraine | 3520-3380 BC |
|  | X17 | MAY10 | FJ204363 | Mayaki, Ukraine | 3650-3500 BC |
| EARLY BRONZE AGE | X9 | Kan3 | FJ204353 | Turkey | 3850 BC |
|  | D2f | Kan5 | FJ204349 | Turkey | 3850 BC |
| BRONZE AGE | X5a | Gar3 | FJ204366 | Garbovat, Romania | 1500-1000 BC |
|  | X3c1a | Gar4 | FJ204367 | Garbovat, Romania | 1500-1000 BC |
|  | X7a | Lch 1 | FJ204370 | Lchashen, Armenia | 1410-1250 BC |
|  | D1 | Lor3 | FJ204350 | Lori-Berd, North Armenia | 1950-1750 BC |
|  | D2 | Lor2 | FJ204368 | Lori-Berd, North Armenia | 1950-1750 BC |
|  | D2 | Lor 1 | FJ204371 | Lori-Berd, North Armenia | 1950-1750 BC |
|  | I | Mic1 | FJ204372 | Miciurin, Moldova | 1500-1000 BC |
|  | D | Mic2 | FJ204373 | Miciurin, Moldova | 1500-1000 BC |
|  | X2b | Mic3 | FJ204374 | Miciurin, Moldova | 1500-1000 BC |
|  | K2b | Mic4 | FJ204375 | Miciurin, Moldova | 1500-1000 BC |
|  | F | Mic5 | FJ204376 | Miciurin, Moldova | 1500-1000 BC |
|  | G4a | MOH1 | FJ204365 | Armenia | Bronze Age |

| **Europe, Armenia** | |  |  |  |  |
| --- | --- | --- | --- | --- | --- |
| IRON AGE | B1b |  | AF326679 | Southern Sweden | 200-500 AD |
|  | X1 |  | AY129530 | Pompeii, Italy | 79 BC |
|  | X7a2 |  | AY129532 | Herculaneum, Italy | 79 BC |
|  | X13 |  | AF326678 | Southern Sweden | 100 BC |
|  | X2 |  | AF326677 | Southern Sweden | 200-500 AD |
|  | B1 |  | AF326676 | Southern Sweden | 200-500 AD |
|  | D2e |  | DQ327848 | Clare, Edenvale, Ireland | 1595 BP |
|  | D3 | Shi 1 | FJ204369 | Shirakavan, Armenia | 895-795 BC |
|  |  |  |  |  |  |
| **Europe (Hungary, United Kingdom)** | | | |  |  |
| MEDIEVAL | I |  | EU559581 | Carpathian Basin Hungary | 900 AD |
|  | I1 |  | EU559580 | Carpathian Basin Hungary | 900 AD |
|  | A |  | EU559582 | Carpathian Basin Hungary | 900 AD |
|  | B1 |  | EU093038 | Carpathian Basin Hungary | 900 AD |
|  | X7 |  | EU093039 | Carpathian Basin Hungary | 900 AD |
|  | X7a1 |  | EU093035 | Carpathian Basin Hungary | 900 AD |
|  | F |  | EU093032 | Carpathian Basin Hungary | 900 AD |
|  | K2b |  | EU093031 | Carpathian Basin Hungary | 900 AD |
|  | K2b |  | EU093033 | Carpathian Basin Hungary | 900 AD |
|  | X16 |  | EU559583 | Carpathian Basin Hungary | 900 AD |
|  | D2a |  | EU093042 | Carpathian Basin Hungary | 900 AD |
|  | D2c |  | EU093041 | Carpathian Basin Hungary | 900 AD |
|  | D3 |  | EU093044 | Carpathian Basin Hungary | 900 AD |
|  | D3 |  | EU093037 | Carpathian Basin Hungary | 900 AD |
|  | D3 |  | EU093030 | Carpathian Basin Hungary | 900 AD |
|  | D3 |  | EU093036 | Carpathian Basin Hungary | 900 AD |
|  | X2a |  | EU093034 | Carpathian Basin Hungary | 900 AD |
|  | X3b |  | EU093040 | Carpathian Basin Hungary | 900 AD |
|  | X2c |  | EU559576 | Carpathian Basin Hungary | 900 AD |
|  | X2b |  | EU559578 | Carpathian Basin Hungary | 900 AD |
|  | X2 |  | EU559579 | Carpathian Basin Hungary | 900 AD |
|  | X2 |  | EU559577 | Carpathian Basin Hungary | 900 AD |
|  | X3c1 |  | EU559575 | Carpathian Basin Hungary | 900 AD |
|  | X3c1 |  | EU559585 | Carpathian Basin Hungary | 600 AD |
|  | X3c1 |  | EU093043 | Carpathian Basin Hungary | 900 AD |
|  | I |  | EU559584 | Carpathian Basin Hungary | 600-700 AD |
|  | X2 |  | DQ327851 | Derbyshire, Carsington Pasture, United Kingdom | 692 AD |

| **Iberian Peninsula** | | |  |  |  |  |
| --- | --- | --- | --- | --- | --- | --- |
| MESOLITHIC-NEOLITHIC | H1 | | 44 | FJ204384 | Atxoste, Iberian Peninsula (Spain) | 5500-4950 BC |
|  | H1 | | 45 | FJ204380 | Atxoste, Iberian Peninsula (Spain) | 5500-4950 BC |
|  | J | | 1 | FJ204390 | Cueva Fosca -Valencia-Cartellon, Iberian Peninsula (Spain) | 5200 -4900BC |
|  | H1 | | 2 | HM802276 | Cueva Fosca -Valencia-Cartellon, Iberian Peninsula (Spain) | 5200 -4900BC |
|  | H1 | | 3 | FJ204381 | Cueva Fosca -Valencia-Cartellon, Iberian Peninsula (Spain) | 5200-4900 BC |
|  | B | | 31 | FJ204382 | Cueva Fosca -Valencia-Cartellon, Iberian Peninsula (Spain) | 5210-4910 BC |
|  | J | | 32 | FJ204383 | Cueva Fosca -Valencia-Cartellon, Iberian Peninsula (Spain) | 5220-4980 BC |
|  | J | | 35 | HM802280 | Cueva Fosca -Valencia-Cartellon, Iberian Peninsula (Spain) | 5380-5210BC |
|  | H1 | | 37 | FJ204385 | Cueva Fosca -Valencia-Cartellon, Iberian Peninsula (Spain) | 5210-4910 BC |
|  | H1 | | 17 | FJ204386 | Cueva De La Vaquera-Segovia, Iberian Peninsula (Spain) | 5210-4940 BC |
| COPPER AGE | | H1 | 27 | HM802281 | El Caprichio-Madrid, Iberian Peninsula (Spain) | 4300-2200 BC |
|  |  | H1 | 20 | HM802277 | Cueva Rubia-Valmayor/Madrid, Iberian Peninsula (Spain) | 2880-2570 BC |
|  |  | A | 21 | HM802278 | Cueva Rubia-Valmayor/Madrid, Iberian Peninsula (Spain) | 2900- 2500 BC |
| BRONZE AGE | | H1 | 23 | HM802279 | Cueva Rubia-Valmayor/Madrid, Iberian Peninsula (Spain) | 1350 BC |
|  |  | H1 | 24 | FJ204389 | Cueva Rubia-Valmayor/Madrid, Iberian Peninsula (Spain) | 1350 BC |
|  |  | H1 |  | DQ683538 | Portalon, Iberian Peninsula (Spain) | 2130-2080 BC |
|  |  | H1 |  | DQ683544 | Portalon, Iberian Peninsula (Spain) | 2480-2290 BC |
|  |  | H1 |  | DQ683543 | Portalon, Iberian Peninsula (Spain) | Bronze Age |
|  |  | H1 |  | DQ683542 | Portalon, Iberian Peninsula (Spain) | Bronze Age |
|  |  | H1 |  | DQ683537 | Portalon, Iberian Peninsula (Spain) | Bronze Age |
|  |  | H1 |  | DQ683536 | Portalon, Iberian Peninsula (Spain) | 2310-2030 BC |
|  |  | J |  | DQ683539 | Portalon, Iberian Peninsula (Spain) | Bronze Age |
|  |  | X4a |  | DQ683532 | Portalon, Iberian Peninsula (Spain) | 2200-1960 BC |
|  |  | B |  | DQ683528 | Portalon, Iberian Peninsula (Spain) | 2200-1970 BC |
|  |  | D2 |  | DQ683526 | Portalon, Iberian Peninsula (Spain) | 1750-1590 BC |
|  |  | A |  | DQ683535 | Portalon, Iberian Peninsula (Spain) | Bronze Age |
|  |  | A |  | DQ683534 | Portalon, Iberian Peninsula (Spain) | 1890-1680 BC |
|  |  | H1 |  | DQ683533 | Portalon, Iberian Peninsula (Spain) | 2040-1880 BC |
|  |  | H1b |  | DQ683530 | Portalon, Iberian Peninsula (Spain) | 2300-2120 BC |
|  |  | H1 |  | DQ683529 | Portalon, Iberian Peninsula (Spain) | 2200-1970 BC |
|  |  | H1a |  | DQ683527 | Portalon, Iberian Peninsula (Spain) | 1920-1720 BC |
|  |  | H1 |  | DQ683540 | Portalon, Iberian Peninsula (Spain) | Bronze Age |
|  |  | H1 |  | DQ683541 | Portalon, Iberian Peninsula (Spain) | Bronze Age |
|  |  | H1b |  | DQ683525 | Portalon, Iberian Peninsula (Spain) | 2130-1900BC |
|  |  | H1 | 39 | FJ204387 | El Acequion, Iberian Peninsula (Spain) | 2200-800 BC |
|  |  | H1 | 40 | FJ204388 | El Acequion, Iberian Peninsula (Spain) | 2200-800 BC |
| IRON AGE | | B | 4 | FJ204391 | Soto de Medinilla -Valladolid, Iberian Peninsula (Spain) | 800 BC - 6 AD |
| MEDIEVAL | | X2 |  | DQ683531 | Iberian Peninsula (Spain) | 980-1050 AD |
|  |  | X2b | 29 | FJ204392 | Mucientes-Valladolid, Iberian Peninsula (Spain) | 660-780 AD |
