# Supplementary material for: Origin and History of Mitochondrial DNA Lineages in Domestic Horses
Source: PLoS One. 2010 Dec 20;5(12):e15311. doi: 10.1371/journal.pone.0015311 (PMC3004868; doi:10.1371/journal.pone.0015311)
Supplement: Table S4 — Variable positions of the d-loop sequences of ancient haplotypes. Nps less 15000 are shown. D2e corresponds to mtDNA reference, X79547 [23]. Mutational hotspots (15585, 15597, 15604 and 15650) are not included, because these positions were excluded in phylogenetic reconstructions. Gaps were signed by “-“. Previous nomenclature [13]–[14] is given in brackets. (DOC) [file pone.0015311.s004.doc]

| *Haplotype name* | ***Nucleotide position in relation to the reference sequence* D2e (A5)** |
| --- | --- |
| **A** (A6) | 495C 602T 720A |
| **B** | 495C 602T 617C 720A |
| **B1** (C1) | 495C 602T 617C 659C 720A |
| **B1a** | 495C 511A 602T 617C 659C 720A |
| **B1b** | 495C 602T 617C 626G 659C 720A |
| **B2** | 495C 602T 617C 671A 720A |
| **B3** | 495C 602T 617C 649G 720A |
| **C** | 495C 602T 649G 720A |
| **C1** | 495C 602T 635T 649G 720A |
| **D** | 495C 720A |
| **D1** | 495C 720A 728- |
| **D2** (A4) | 495C |
| **D2a** | 495C 539T |
| **D2b** | 495C 605T |
| **D2c** | 495C 657C |
| **D2d** | 495C 723T |
| **D2e** (A5) | *Reference mtDNA sequence* |
| **D2f** | 495C 584T |
| **D3** (A3) | 495C 666A 720A |
| **D3a** | 495C 666A 703C 720A |
| **E** | 495C 532- 602T 720A |
| **E1** | 495C 532- 600A 602T 720A |
| **F** (C2) | 495C 601C 602T 720A |
| **G** | 495C 521A 595G 602T 720A |
| **G1** | 495C 521A 596G 602T 720A |
| **G2** | 495C 595G 602T 720A |
| **G3** (E) | 495C 521A 602T 720A 736C |
| **G4a** | 495C 521A 596G 602T 711T 720A 736C |
| **Gx4** | 495C 521A 596G 602T 720A 736C |
| **H** | 495C 536C 602T 720A |
| **H1** | 495C 533- 536C 602T 720A |
| **H1a** | 495C 533- 536C 600A 602T 720A |
| **H1b** | 495C 533- 536C 602T 697C 720A |
| **I** (B2) | 495C 538G 602T 709T 720A |
| **I1** (B1) | 495C 538G 596G 602T 709T 720A |
| **I2a** | 495C 507A 533G 538G 602T 709T 720A |
| **J** | 495C 602T 718T 720A |
| **K** | 495C 602T 703C 720A |
| **K1** | 495C 602T 666A 703C 720A |
| **K2** (F3) | 495C 602T 703C 720A 740G |
| **K2a** | 495C 595G 602T 703C 720A 740G |
| **K2b** (F2) | 495C 602T 703C 720A 726A 740G |
| **K2b1** | 495C 602T 659C 703C 720A 726A 740G |
| **K2b2** | 495C 602T 660G 703C 718T 720A 726A 740G |
| **K3** (F1) | 495C 602T 667G 703C 720A |
| **K3a** | 495C 602T 635T 667G703C 720A |
| **K3a1** | 495C 602T 635T 667G703C 718T 720A |
| **K3b** | 495C 602T 632C 667G 703C 720A |
| **X1** | 534T 602T 603C 649G 653G 720A |
| **X2** (D1/D3) | 494C 495C 496G 534T 602T 603C 649G 720A |
| **X2a** | 494C 495C 496G 534T 602T 603C 649G 703C 720A |
| **X2b** (D2) | 494C 495C 496G 534T 603C 649G 720A |
| **X2c** | 494C 495C 496G 534T 583G 602T 603C 623C 649G 720A |
| **X2d** | 494C 495C 496G 602T 603C 666A 720A |
| **X3** (A7) | 495C 542T 602T 666A 720A |
| **X3a** | 495C 542T 602T 666A 684A 720A |
| **X3b** | 495C 542T 602T 626G 666A 720A |
| **X3c** | 495C 542T 602T 635T 666A 720A |
| **X3c1** (A1) | 495C 542T 602T 635T 666A 703C 720A |
| **X3c1a** | 495C 542T 602T 635T 703C 720A |
| **X3c2** | 542T 602T 666A 709T 720A |
| **X3d** | 495C 542T 602T 651A 666A 720A |
| **X4** | 495C 526C 540G 602T 718T 720A |
| **X4a** | 495C 526C 540G 602T 649G 718T 720A |
| **X5** | 495C 544C 602T 635T 686G 720A |
| **X5a** | 495C 544C 595G 602T 635T 686G 720A |
| **X6** | 495C 526C 602T 635T 703C 720A |
| **X6a** | 495C 526C 533T 602T 635T 703C 720A |
| **X6b** | 495C 526C 544C 602T 635T 703C 720A |
| **X6c** | 495C 526C 602T 635T 659C 703C 720A |
| **X7** | 495C 598C 602T 615G 659C 703C 720A |
| **X7a** | 495C 598C 602T 615G 616G 659C 703C 720A |
| **X7a1** | 495C 598C 602T 615G 616G 703C 720A |
| **X7a2** | 598C 602T 615G 616G 659C 669T 703C 720A |
| **X7a3** | 495C 602T 615G 616G 659C 703C 720A |
| **X7a4** | 495C 598C 602T 616G 659C 703C 720A |
| **X8** | 495C 602T 617C 703C 709T 720A |
| **X8a** | 495C 602T 617C 703C 720A |
| **X9** | 495C 602T 687A 703C 717T 720A |
| **X10** | 495C 570A 602T 635T 703C 720A |
| **X11** | 495C 526C 566C 602T 649G 720A |
| **X12** | 495C 526C *Insertion of* C *between* 532-533 546T 602T 617C 649G 703C 720A |
| **X13** | 495C 526C 528T 541T 602T 635T 666A 703C 720A |
| **X14** | 498T 526C 576T 577T 602T 659C 703C 716G 720A |
| **X15** | 495C 544C 558A 602T 720A |
| **X16** | 495C 509T 510A 567G 703C 720A 740G |
| **X17** | 495C 561T 598C 602T 720A 726A |
