# Supplementary figures and images for: Origin and History of Mitochondrial DNA Lineages in Domestic Horses
Source: PLoS One. 2010 Dec 20;5(12):e15311. doi: 10.1371/journal.pone.0015311 (PMC3004868; doi:10.1371/journal.pone.0015311)

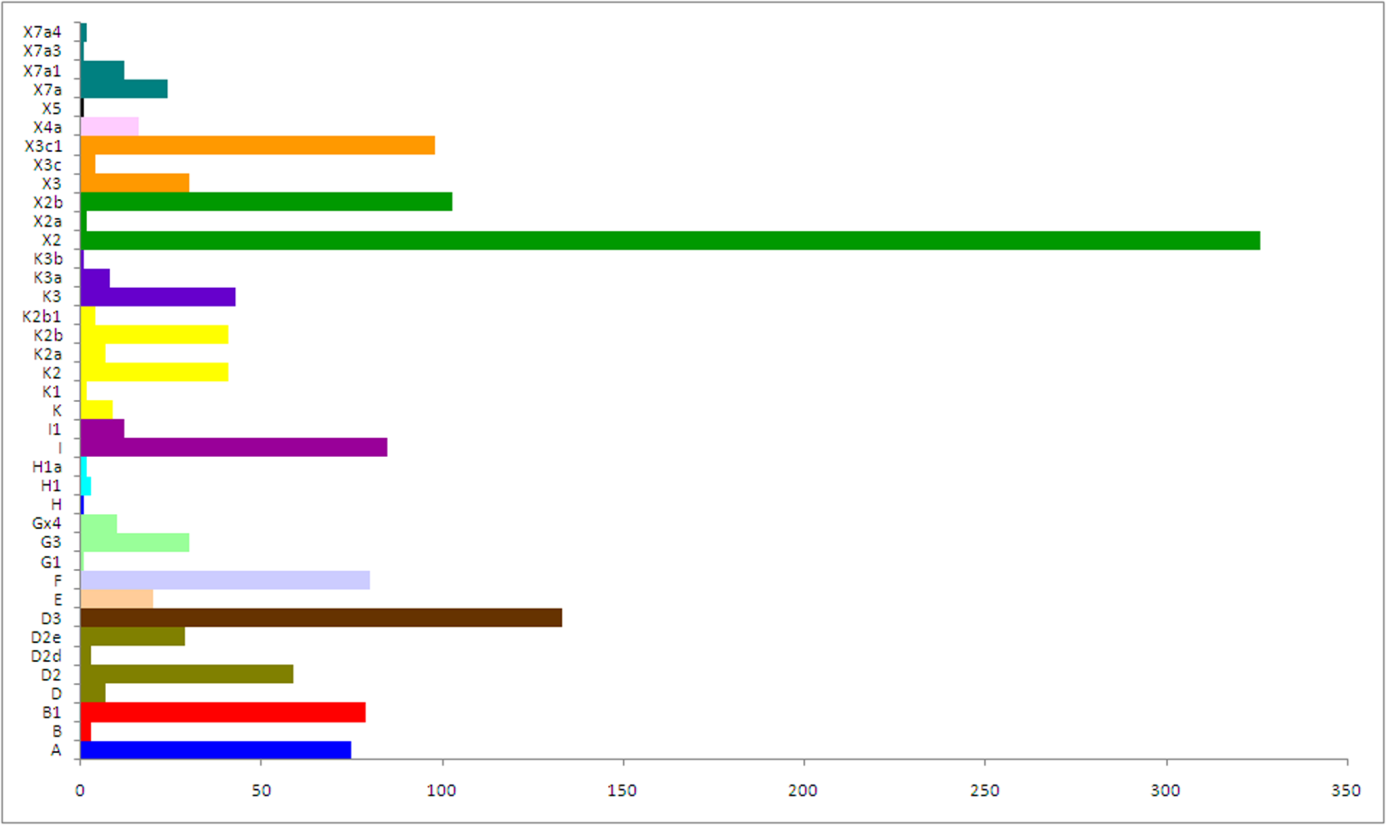

Supplement: Table S6 — Occurrence of ancient haplotypes in modern horses based on their Genbank entries. (DOC) [file pone.0015311.s006.doc]
