# Supplementary material for: Origin and History of Mitochondrial DNA Lineages in Domestic Horses
Source: PLoS One. 2010 Dec 20;5(12):e15311. doi: 10.1371/journal.pone.0015311 (PMC3004868; doi:10.1371/journal.pone.0015311)
Supplement: Table S7 — Sum of squared deviation and raggedness index (mismatch distribution). (DOC) [file pone.0015311.s007.doc]

| *4.350-3.000BC Europe/Asia Minor/Armenia* | Sum of Squared deviation (SSD): 0.068 |
| --- | --- |
|  | P(SSD): 0.050 |
|  | Raggedness index (r): 0.119 |
|  | P(r): 0.070 |
| *5.500-3.000BC Iberian Peninsula* | Sum of Squared deviation: 0.374 |
|  | P(SSD): 0.000 |
|  | Raggedness index: 0.738 |
|  | P(r): 0.000 |
| *2.000BC-600AD China* | Sum of Squared deviation: 0.012 |
|  | P(SSD): 0.084 |
|  | Raggedness index: 0.051 |
|  | P(r): 0.022 |
| *3.000BC-600AD West/South Siberia, Kazakhstan* | Sum of Squared deviation: 0.003 |
|  | P(SSD): 0.586 |
|  | Raggedness index: 0.012 |
|  | P(r): 0.723 |
| *3.000BC-600AD Europe/Armenia* | Sum of Squared deviation: 0.003 |
|  | P(SSD): 0.740 |
|  | Raggedness index: 0.011 |
|  | P(r): 0.882 |
| *3.000BC-600AD Iberian Peninsula* | Sum of Squared deviation: 0.080 |
|  | P(SSD): 0.296 |
|  | Raggedness index: 0.083 |
|  | P(r): 0.730 |
| *Early Medieval Europe (Iberia excluded)* | Sum of Squared deviation: 0.002 |
|  | P(SSD): 0.823 |
|  | Raggedness index: 0.008 |
|  | P(r): 0.922 |
