# Supplementary material for: Origin and History of Mitochondrial DNA Lineages in Domestic Horses
Source: PLoS One. 2010 Dec 20;5(12):e15311. doi: 10.1371/journal.pone.0015311 (PMC3004868; doi:10.1371/journal.pone.0015311)
Supplement: Table S10 — 14C Dates of samples analyzed in this study. For details of samples see table 1. The Pleistocene samples are not directly dated, but estimated from context should be around 20,000 years old. The remaining dates are either calibrated carbon dates or derived from archaeological context. (DOC) [file pone.0015311.s010.doc]

| **Sample** | **Accession** | **Age ^14^C** | **Calibration date** | **Lab. No.** | **Detail** |
| --- | --- | --- | --- | --- | --- |
| Fen 1 | FJ204377 | 2695 ± 30 BP | cal. 905-800 BC | Poz-22612 | 3.4%N 11.0%C |
| Spa 1 | FJ204354 |  | cal. 9390-9210 BC |  |  |
| VIT2 | FJ204357 | 5400 ± 40 BP | cal. 4350-4220 BC | Poz-24899 | 1.5%N 6.0%C |
| VIT4 |  | 5430 ± 40 BP | cal. 4360-4220 BC | Poz-24900 | 1.7%N 6.5%C |
| CAS1 | FJ204356 | 4820 ± 40 BP | cal. 3700-3380 BC | Poz-24925 | 1.0%N 7.6%C carbonate |
| MAY3 |  | 4745 ± 35 BP | cal. 3640-3490 BC | Poz-24926 | 0.6%N 2.8%C carbonate |
| MAY5 | FJ204360 | 4550 ± 35 BP | cal. 3250-3100 BC | Poz-24826 | 2.4%N 9.8%C |
| MAY6 | FJ204361 | 4605 ± 35 BP | cal. 3520-3330 BC | Poz-24849 | 1.6%N 6.7%C |
| MAY7 | FJ204362 | 4640 ± 35 BP | cal. 3520-3380 BC | Poz-24850 | 2.6%N 10.4%C |
| MAY10 | FJ204363 | 4770 ± 40 BP | cal. 3650-3500 BC | Poz-24927 | 0.5%N 3.4%C carbonate |
| Lch 1 | FJ204370 | 3050 ± 30 BP | cal. 1410-1250 BC | Poz-22613 | 3.7%N 12.4%C |
| Lor 1 | FJ204371 | 3525 ± 35 BP | cal. 1950-1750 BC | Poz-22701 | 1.1%N 4.6%C |
| Shi 1 | FJ204369 | 2670 ± 30 BP | cal. 895-795 BC | Poz-22615 | 1.5%N 5.5%C |
| 31 | FJ204382 | 6100 ± 40 BP | cal. 5210-4910 BC | Poz-24720 | 3.5%N 15.4%C |
| 32 | FJ204383 | 6135 ± 35 BP | cal. 5220-4980 BC | Poz-24743 | 3.4%N 17.2%C |
| 37 | FJ204385 | 6100 ± 40 BP | cal. 5210-4910 BC | Poz-24745 | 2.6%N 11.9%C |
| 17 | FJ204386 | 6110 ± 40 BP | cal. 5210-4940 BC | Poz-24721 | 2.7%N 13.9%C |
| 29 | FJ204392 | 1275 ± 25 BP | cal. 660-780 AD | Poz-24740 | 1.2%N 6.9%C |
| 30 |  | 1230 ± 30 BP | cal. 680-890AD | Poz-24739 | 3.4%N 15%C |
