# Supplementary material for: Origin and History of Mitochondrial DNA Lineages in Domestic Horses
Source: PLoS One. 2010 Dec 20;5(12):e15311. doi: 10.1371/journal.pone.0015311 (PMC3004868; doi:10.1371/journal.pone.0015311)
Supplement: Table S11 — GenBank Accession no of modern horses (Przewalski horses are bold). (DOC) [file pone.0015311.s011.doc]

AB329587, AB329588, AB329589, AB329590, AB329591, AB329592, AB329593, AB329594, AB329595, AB329596, AB329597, AB329598, AB329599, AB329600, AB329601, AB329602, AB329603, AB329604, AB329605, AB329606, AB329607, AB329608, AB329609, AB329610, AB329611, AB329612, AB329613, AB329614, AB329615, AB329616, AB329617, AB329618, AB329619, AB329620, AB329621, AB329622, AB329623, AB329624, AB329625, AB329626, AB329627, AB329628, AF014405, AF014406, AF014407, AF014408, **AF014409**, AF014410, AF014411, AF014412, AF014413, AF014414, AF014415, AF014416, AF014417, AF056071, AF064627, AF064628, AF064629, AF064630, AF064632, AF072975, AF072976, AF072977, AF072978, AF072980, AF072981, AF072982, AF072984, AF072985, AF072987, AF072988, AF072989, AF072990, AF072991, AF072992, AF072993, **AF072995**, AF072996, AF132568, AF132569, AF132570, AF132571, AF132572, AF132573, AF132574, AF132575, AF132576, AF132577, AF132578, AF132580, AF132582, AF132583, AF132584, AF132585, AF132586, AF132587, AF132588, AF132589, AF132590, AF132591, AF132592, AF132593, AF132594, AF168689, AF168690, AF168691, AF168692, AF168693, AF168694, AF168695, AF168696, AF168697, AF168698, AF326635, AF326636, AF326637, AF326638, AF326639, AF326640, AF326641, AF326642, AF326643, AF326644, AF326645, AF326646, AF326647, AF326648, AF326649, AF326650, AF326651, AF326652, AF326653, AF326654, AF326655, AF326656, AF326657, AF326658, AF326659, AF326660, AF326661, AF326662, AF326663, AF326664, AF326665, AF326666, AF326667, AF354425, AF354426, AF354427, AF354428, AF354429, AF354430, AF354431, AF354432, AF354433, AF354434, AF354435, AF354436, AF354437, AF354438, AF354439, AF354440, AF354441, AF431965, AF431966, AF431967, AF431968, AF431969, AF447764, AF447765, AF465984, AF465985, AF465986, AF465987, AF465988, AF465989, AF465990, AF465991, AF465992, AF465993, AF465994, AF465995, AF465996, AF465997, AF465998, AF465999, AF466000, AF466001, AF466002, AF466003, AF466004, AF466005, AF466006, AF466007, AF466008, AF466009, AF466010, AF466011, AF466012, AF466013, AF466014, AF466015, AF466016, AF481232, AF481233, AF481234, AF481235, AF481236, AF481237, AF481238, AF481239, AF481240, AF481241, AF481242, AF481243, AF481244, AF481245, AF481246, AF481247, AF481248, AF481249, AF481250, AF481251, AF481252, AF481253, AF481254, AF481255, AF481256, AF481257, AF481258, AF481259, AF481260, AF481261, AF481262, AF481263, AF481264, AF481265, AF481266, AF481267, AF481268, AF481269, AF481270, AF481271, AF481272, AF481273, AF481274, AF481275, AF481276, AF481277, AF481278, AF481279, AF481280, AF481281, AF481282, AF481283, AF481284, AF481285, AF481286, AF481287, AF481288, AF481289, AF481290, AF481291, AF481292, AF481293, AF481294, AF481295, AF481296, AF481297, AF481298, AF481299, AF481300, AF481301, AF481302, AF481303, AF481304, AF481305, AF481306, AF481307, AF481308, AF481309, AF481310, AF481311, AF481312, AF481313, AF481314, AF481315, AF481316, AF481317, AF481318, AF481319, AF481320, AF481321, AF481322, AF481323, AF481324, AF481325, AF481326, AF481327, AF481328, AF481329, AF481330, AF481331, AF481332, AF481333, AF481334, AF516489, AF516490, AF516491, AF516492, AF516493, AF516494, AF516495, AF516496, AF516497, AF516498, AF516499, AF516500, AF516501, AF516502, AF516503, AF516504, AF516505, AF516506, AF516507, AF516508, AF516509, AF516510, AF516511, AJ413608, AJ413609, AJ413610, AJ413611, AJ413612, AJ413613, AJ413614, AJ413615, AJ413616, AJ413617, AJ413618, AJ413619, AJ413620, AJ413621, AJ413622, AJ413623, AJ413624, AJ413625, AJ413626, AJ413627, AJ413628, AJ413629, AJ413630, AJ413631, AJ413632, AJ413633, AJ413634, AJ413635, AJ413636, AJ413637, AJ413638, AJ413639, AJ413640, AJ413641, AJ413642, AJ413643, AJ413644, AJ413645, AJ413646, AJ413647, AJ413648, AJ413649, AJ413650, AJ413651, AJ413652, AJ413653, AJ413654, AJ413655, AJ413656, AJ413657, AJ413658, AJ413659, AJ413660, AJ413661, AJ413662, AJ413663, AJ413664, AJ413665, AJ413666, AJ413667, AJ413668, AJ413669, AJ413670, AJ413671, AJ413672, AJ413673, AJ413674, AJ413675, AJ413676, AJ413677, AJ413678, AJ413679, AJ413680, AJ413681, AJ413682, AJ413683, AJ413684, AJ413685, AJ413686, AJ413687, AJ413688, AJ413689, AJ413690, AJ413691, AJ413692, AJ413693, AJ413694, AJ413695, AJ413696, AJ413697, AJ413698, AJ413699, AJ413700, AJ413701, AJ413702, AJ413703, AJ413704, AJ413705, AJ413706, AJ413707, AJ413708, AJ413709, AJ413710, AJ413711, AJ413712, AJ413713, AJ413714, AJ413715, AJ413716, AJ413717, AJ413718, AJ413719, AJ413720, AJ413721, AJ413722, AJ413723, AJ413724, AJ413725, AJ413726, AJ413727, AJ413728, AJ413729, AJ413730, AJ413731, AJ413732, AJ413733, AJ413734, AJ413735, AJ413736, AJ413737, AJ413738, AJ413739, AJ413740, AJ413741, AJ413742, AJ413743, AJ413744, AJ413745, AJ413746, AJ413747, AJ413748, AJ413749, AJ413750, AJ413751, AJ413752, AJ413753, AJ413754, AJ413755, AJ413756, AJ413757, AJ413758, AJ413759, AJ413760, AJ413761, AJ413762, AJ413763, AJ413764, AJ413765, AJ413766, AJ413767, AJ413768, AJ413769, AJ413770, AJ413771, AJ413772, AJ413773, AJ413774, AJ413775, AJ413776, AJ413777, AJ413778, AJ413779, AJ413780, AJ413781, AJ413782, AJ413783, AJ413784, AJ413785, AJ413786, AJ413787, AJ413788, AJ413789, AJ413790, AJ413791, AJ413792, AJ413793, AJ413794, AJ413795, AJ413796, AJ413797, AJ413798, AJ413799, AJ413800, AJ413801, AJ413802, AJ413803, AJ413804, AJ413805, AJ413806, AJ413807, AJ413808, AJ413809, AJ413810, AJ413811, AJ413812, AJ413813, AJ413814, AJ413815, AJ413816, AJ413817, AJ413818, AJ413819, AJ413820, AJ413821, AJ413822, AJ413823, AJ413824, AJ413825, AJ413826, AJ413827, AJ413828, AJ413829, AJ413830, AJ413831, AJ413832, AJ413833, AJ413834, AJ413835, AJ413836, AJ413837, AJ413838, AJ413839, AJ413840, AJ413841, AJ413842, AJ413843, AJ413844, AJ413845, AJ413846, AJ413847, AJ413848, AJ413849, AJ413850, AJ413851, AJ413852, AJ413853, AJ413854, AJ413855, AJ413856, AJ413857, AJ413858, AJ413859, AJ413860, AJ413861, AJ413862, AJ413863, AJ413864, AJ413865, AJ413866, AJ413867, AJ413868, AJ413869, AJ413870, AJ413871, AJ413872, AJ413873, AJ413874, AJ413875, AJ413876, AJ413877, AJ413878, AJ413879, AJ413880, AJ413881, AJ413882, AJ413883, AJ413884, AJ413885, AJ413886, AJ413887, AJ413888, AJ413889, AJ413890, AJ413891, AJ413892, AJ413893, AJ413894, AJ413895, AJ413896, AJ413897, AJ413898, AJ413899, AJ413900, AJ413901, AJ413902, AJ413903, AJ413904, AJ413905, AJ413906, AJ413907, AJ413908, AJ413909, AJ413910, AJ413911, AJ413912, AJ413913, AJ413914, AJ413915, AJ413916, AJ413917, AJ413918, AJ413919, AJ413920, AJ413921, AJ413922, AJ413923, AJ413924, AJ413925, AJ413926, AY049718, AY049719, AY057408, AY057409, AY057410, AY057411, AY057412, AY057413, AY057414, AY057415, AY057416, AY057417, AY057418, AY057419, AY057420, AY057421, AY057422, AY057423, AY057424, AY057425, AY057426, AY057427, AY057428, AY057429, AY057430, AY057431, AY057432, AY057433, AY057434, AY136785, AY136786, AY246174, AY246175, AY246176, AY246177, AY246178, AY246179, AY246180, AY246181, AY246182, AY246183, AY246184, AY246185, AY246186, AY246187, AY246188, AY246189, AY246190, AY246191, AY246192, AY246193, AY246194, AY246195, AY246196, AY246197, AY246198, AY246199, AY246200, AY246201, AY246202, AY246203, AY246204, AY246205, AY246206, AY246207, AY246208, AY246209, AY246210, AY246211, AY246212, AY246213, AY246214, AY246215, AY246216, AY246217, AY246218, AY246219, AY246220, AY246221, AY246222, AY246223, AY246224, AY246225, AY246226, AY246227, AY246228, AY246229, AY246230, AY246231, AY246232, AY246233, AY246234, AY246235, AY246236, AY246237, AY246238, AY246239, AY246240, AY246241, AY246242, AY246243, AY246244, AY246245, AY246246, AY246247, AY246248, AY246249, AY246250, AY246251, AY246252, AY246253, AY246254, AY246255, AY246256, AY246257, AY246258, AY246259, AY246260, AY246261, AY246262, AY246263, AY246264, AY246265, AY246266, AY246267, AY246268, AY246269, AY246270, AY246271, AY293975, AY293976, AY293977, AY293978, AY293979, AY293980, AY293981, AY293982, AY293983, AY293984, AY293985, AY293986, AY293987, AY293988, AY293989, AY293990, AY293991, AY462421, AY462422, AY462423, AY462424, AY462425, AY462426, AY462427, AY462428, AY462429, AY462430, AY462431, AY462432, AY462433, AY462434, AY462435, AY462436, AY462437, AY462438, AY462439, AY462440, AY462441, AY462442, AY462443, AY462444, AY462445, AY462446, AY462447, AY462448, AY462449, AY462450, AY462451, AY462452, AY462453, AY462454, AY462455, AY519871, AY519872, AY519873, AY519874, AY519875, AY519876, AY519877, AY519878, AY519879, AY519880, AY519881, AY519882, AY519883, AY519884, AY519885, AY519886, AY519887, AY519888, AY519889, AY519890, AY519891, AY519892, AY519893, AY519894, AY519895, AY519896, AY519897, AY519898, AY519899, AY519900, AY519901, AY519902, AY519903, AY519904, AY519905, AY519906, AY519907, AY519908, AY519909, AY519910, AY519911, AY519912, AY519913, AY519914, AY519915, AY519916, AY519917, AY519918, AY519919, AY519920, AY519921, AY519922, AY519923, AY519924, AY519925, AY519926, AY519927, AY519928, AY519929, AY519930, AY519931, AY519932, AY519933, AY519934, AY519935, AY519936, AY519937, AY519938, AY519939, AY519940, AY519941, AY519942, AY519943, AY519944, AY519945, AY519946, AY519947, AY519948, AY519949, AY519950, AY519951, AY519952, AY519953, AY519954, AY519955, AY519956, AY519957, AY519958, AY519959, AY519960, AY519961, AY519962, AY519963, AY519964, AY519965, AY519966, AY519967, AY519968, AY519969, AY519970, AY525091, AY525092, AY525093, AY525094, AY525095, AY525096, AY569548, AY569549, AY569550, AY575103, AY575104, AY575105, AY575106, AY575107, AY575108, AY575109, AY575110, AY575111, AY575112, AY575113, AY575114, AY575115, AY575116, AY575117, AY575118, AY575119, AY575120, AY575121, AY575122, AY575123, AY575124, AY575125, AY575126, AY575127, AY575128, AY575129, AY575130, AY575131, AY575132, AY575133, AY575134, AY575135, AY575136, AY575137, AY575138, AY575139, AY584828, AY805641, AY805642, AY805643, AY805644, AY805645, AY805646, AY805647, AY805648, AY805649, AY805650, AY805651, AY805652, AY805653, AY805654, AY805655, AY805656, AY805657, AY805658, AY805659, AY805660, AY805661, AY805662, AY805663, AY805664, AY997128, AY997129, AY997130, AY997131, AY997132, AY997133, AY997134, AY997135, AY997136, AY997137, AY997138, AY997139, AY997140, AY997141, AY997142, AY997143, AY997144, AY997145, AY997146, AY997147, AY997148, AY997149, AY997150, AY997151, AY997152, AY997153, AY997154, AY997155, AY997156, AY997157, AY997158, AY997159, AY997160, AY997161, AY997162, AY997163, AY997164, AY997165, AY997166, AY997167, AY997168, AY997169, AY997170, AY997171, AY997172, AY997173, AY997174, AY997175, AY997176, AY997177, AY997178, AY997179, AY997180, AY997181, AY997182, AY997183, AY997184, AY997185, AY997186, AY997187, AY997188, AY997189, AY997190, AY997191, AY997192, AY997193, AY997194, AY997195, AY997196, AY997197, AY997198, AY997199, AY997200, AY997201, AY997202, AY997203, AY997204, D14991, D23665, D23666, DQ233731, DQ233732, DQ297622, DQ297623, DQ297624, DQ297625, DQ297626, DQ297627, DQ297628, DQ297629, DQ297630, DQ297631, DQ297632, DQ297633, DQ297634, DQ297635, DQ297636, DQ297637, DQ297638, DQ324048, DQ327838, DQ327839, DQ327840, DQ327841, DQ327842, DQ327843, DQ327844, DQ327845, DQ327846, DQ327847, DQ327852, DQ327853, DQ327854, DQ327855, DQ327856, DQ327857, DQ327858, DQ327859, DQ327860, DQ327861, DQ327862, DQ327863, DQ327864, DQ327865, DQ327866, DQ327867, DQ327868, DQ327869, DQ327870, DQ327871, DQ327872, DQ327873, DQ327874, DQ327875, DQ327876, DQ327877, DQ327878, DQ327879, DQ327880, DQ327881, DQ327882, DQ327883, DQ327884, DQ327885, DQ327886, DQ327887, DQ327888, DQ327889, DQ327890, DQ327891, DQ327892, DQ327893, DQ327894, DQ327895, DQ327896, DQ327897, DQ327898, DQ327899, DQ327900, DQ327901, DQ327902, DQ327903, DQ327904, DQ327905, DQ327906, DQ327907, DQ327908, DQ327909, DQ327910, DQ327911, DQ327912, DQ327913, DQ327914, DQ327915, DQ327916, DQ327917, DQ327918, DQ327919, DQ327920, DQ327921, DQ327922, DQ327923, DQ327924, DQ327925, DQ327926, DQ327927, DQ327928, DQ327929, DQ327930, DQ327931, DQ327932, DQ327933, DQ327934, DQ327935, DQ327936, DQ327937, DQ327938, DQ327939, DQ327940, DQ327941, DQ327942, DQ327943, DQ327944, DQ327945, DQ327946, DQ327947, DQ327948, DQ327949, DQ327950, DQ327951, DQ327952, DQ327953, DQ327954, DQ327955, DQ327956, DQ327957, DQ327958, DQ327959, DQ327960, DQ327961, DQ327962, DQ327963, DQ327964, DQ327965, DQ327966, DQ327967, DQ327968, DQ327969, DQ327970, DQ327971, DQ327972, DQ327973, DQ327974, DQ327975, DQ327976, DQ327977, DQ327978, DQ327979, DQ327980, DQ327981, DQ327982, DQ327983, DQ327984, DQ327985, DQ327986, DQ327987, DQ327988, DQ327989, DQ327990, DQ327991, DQ327992, DQ327993, DQ327994, DQ327995, DQ327996, DQ327997, DQ327998, DQ327999, DQ328000, DQ328001, DQ328002, DQ328003, DQ328004, DQ328005, DQ328006, DQ328007, DQ328008, DQ328009, DQ328010, DQ328011, DQ328012, DQ328013, DQ328014, DQ328015, DQ328016, DQ328017, DQ328018, DQ328019, DQ328020, DQ328021, DQ328022, DQ328023, DQ328024, DQ328025, DQ328026, DQ328027, DQ328028, DQ328029, DQ328030, DQ328031, DQ328032, DQ328033, DQ328034, DQ328035, DQ328036, DQ328037, DQ328038, DQ328039, DQ328040, DQ328041, DQ328042, DQ328043, DQ328044, DQ328045, DQ328046, DQ328047, DQ328048, DQ328049, DQ328050, DQ328051, DQ328052, DQ328053, DQ328054, DQ328055, DQ328056, DQ328057, DQ986464, DQ986465, DQ986466, DQ986467, DQ986468, DQ986469, DQ986470, DQ986471, DQ986472, DQ986473, DQ986474, DQ986475, DQ986476, DQ986477, DQ986478, DQ986479, EF014970, EF014971, EF014972, EF014973, EF014974, EF014975, EF014976, EF014977, EF014978, EF014979, EF014980, EF014981, EF014982, EF014983, EF014984, EF014985, EF014986, EF014987, EF014988, EF014989, EF433374, EF433375, EF433376, EF433377, EF433378, EF433379, EF433380, EF433381, EF433382, EF433383, EF433384, EF433385, EF433386, EF433387, EF433388, EF433389, EF433390, EF433391, EF433392, EF433393, EF433394, EF433395, EF433396, EF433397, EF433398, EF433399, EF433400, EF437553, EF437554, EF437555, EF437556, EF437557, EF437558, EF437559, EF437560, EF437561, EF437562, EF437563, EF437564, EF437565, EF437566, EF597512, EF597513, EF597514, EF686021, EF686022, EF686023, EF686024, EF686025, EF686026, EF686027, EF686028, EF686029, EF686030, EF686031, EF686032, EF686033, EF686034, EF686035, EF686036, EF686037, EF686038, EF686039, EF686040, EF686041, EF686042, EF686043, EF686044, EF686045, EU093045, EU093046, EU093047, EU093048, EU093049, EU093050, EU093051, EU093052, EU093053, EU093054, EU093055, EU093056, EU093057, EU093058, EU093059, EU093060, EU093061, EU093062, EU093063, EU093064, EU093065, EU093066, EU093067, EU093068, EU093069, EU093070, EU093071, EU093072, EU093073, EU256571, EU256572, EU256573, EU256574, EU256575, EU256576, EU256577, EU256578, EU256579, EU256580, EU256581, EU256582, EU256583, EU256584, EU256585, EU256586, EU256587, EU256588, EU256589, EU256590, EU256591, EU256592, EU256593, EU256594, EU256595, EU256596, EU256597, EU256598, EU256599, EU256600, EU256601, EU256602, EU256603, EU256604, EU256605, EU256606, EU256607, EU256608, EU256609, EU256610, EU256611, EU256612, EU256613, EU256614, EU256615, EU256616, EU256617, EU256618, EU256619, EU256620, EU256621, EU256622, EU433687, EU433688, EU433689, EU433690, EU433691, EU433692, EU433693, EU433694, EU433695, EU433696, EU433697, EU433698, EU433699, EU433700, EU433701, EU433702, EU433703, EU433704, EU433705, EU433706, EU433707, EU433708, EU433709, EU433710, EU433711, EU433712, EU433713, EU433714, EU433715, EU433716, EU433717, EU433718, EU433719, EU433720, EU433721, EU433722, EU433723, EU433724, EU433725, EU433726, EU433727, EU433728, EU433729, EU433730, EU433731, EU433732, EU433733, EU433734, EU433735, EU433736, EU433737, EU433738, EU433739, EU433740, EU433741, EU433742, EU433743, EU433744, EU433745, EU433746, EU433747, EU433748, EU433749, EU433750, EU433751, EU433752, EU433753, EU433754, EU433755, EU433756, EU433757, EU433758, EU433759, EU433760, EU433761, EU433762, EU433763, EU433764, EU433765, EU433766, EU433767, EU433768, EU433769, EU433770, EU433771, EU433772, EU433773, EU433774, EU433775, EU433776, EU433777, EU433778, EU433779, EU433780, EU433781, EU433782, EU433783, EU433784, EU433785, EU433786, EU433787, EU433788, EU433789, EU433790, EU433791, EU433792, EU433793, EU433794, EU433795, EU433796, EU433797, EU433798, EU433799, EU433800, EU433801, EU433802, EU433803, EU433804, EU433805, EU433806, EU433807, EU433808, EU433809, EU433810, EU433811, EU433812, EU433813, EU433814, EU433815, EU433816, EU433817, EU433818, EU433819, EU433820, EU433821, EU433822, EU433823, EU433824, EU433825, EU433826, EU433827, EU433828, EU433829, EU433830, EU433831, EU433832, EU433833, EU433834, EU433835, EU433836, EU433837, EU433838, EU433839, EU433840, EU433841, EU433842, EU433843, EU433844, EU433845, EU433846, EU433847, EU433848, EU433849, EU433850, EU569297, EU580148, EU580149, EU580150, EU580151, EU580152, EU580153, EU580154, EU580155, EU580156, EU580157, EU580158, EU580159, EU580160, EU580161, EU580162, EU580163, EU580164, EU580165, EU580166, EU580167, EU580168, EU580169, EU580170, EU580171, EU580172, EU604815, EU604816, EU604817, EU750716, EU750717, EU750718, EU750719, EU750720, EU750721, EU750722, EU750723, EU750724, EU750725, EU750726, EU750727, EU750728, EU750729, EU750730, EU750731, EU826536, EU831234, EU831235, EU831237, EU831238, EU831239, EU939445, FJ392562, FJ392563, FJ392564, FJ392565, FJ392566, FJ392567, FJ392568, FJ392569, FJ392570, FJ392571, FJ392572, FJ392573, FJ392574, FJ392575, FJ392576, FJ392577, FJ392578, FJ392579, FJ392580, FJ502832, FJ502833, FJ502834, FJ502835, FJ502836, FJ502837, FJ502838, FJ502839, FJ624150, FJ624151, FJ624152, FJ624153, FJ624154, FJ624155, FJ624156, FJ624157, NC_001640, X79547.
