# Supplementary material for: Origin and History of Mitochondrial DNA Lineages in Domestic Horses
Source: PLoS One. 2010 Dec 20;5(12):e15311. doi: 10.1371/journal.pone.0015311 (PMC3004868; doi:10.1371/journal.pone.0015311)
Supplement: Supplementary file 8 [file pone.0015311.s003.doc]

	1	2	3	4	5	6	10	12	13	14	15	16	17	18	19	20	21	22	23	24	25	26	28	30	31	32	36	37	38	39	40	42	43	44	45	46	47	48	49	50	51	52	53	54	55	56	57	59	60	63	64	66	68	69	70	72	74	75	77	78	79	82	84	86	88	89	90	91	96	97	98	99	100	108	109	110	111	112	113	114
Position: 15.494-15.740	494	495	496	497	498	499	503	505	506		507	508	509		510		511	512	513	514	515	516	518	520	521	522	526	527	528	529	530	532		533	534	535	536	537	538	539	540	541	542	543	544	545	546	548	549	552	553	555	557	558	559	561	563	564	566		567	570	572	574	576		577	578	583	584	585	586	587	595	596	597	598	599	600	601
Nucleotide sequence of the reference X79547; *Xu and Arnason, 1994* (Insertions red colored)	T	T	A	C	C	C	T	T	G		C	T	A		T		G	T	C	A	G	T	T	A	G	A	T	A	C	C	C	C		A	C	A	T	A	A	C	A	C	C	A	T	A	C	C	A	T	G	C	T	G	C	A	A	T	T		T	G	A	G	C		C	T	A	C	G	T	C	A	A	A	T	T	G	T
Sites with deletions																																																																																	
Monomorphic sites																																																																																	
Polymorphic sites 2 nucleotides																																																																																	
Polymorphic sites 3 nucleotides																																																																																	
Max number of mutations at character	11	10	7	4	1	1	1	1	2	1	2	3	3	2	2	1	3	1	6	2	2	1	2	1	7	1	7	2	3	1	4	12	1	7	10	1	2	2	6	1	7	1	11	2	5	2	1	2	4	1	1	1	1	2	2	2	2	1	2	1	2	2	1	3	2	1	1	1	2	5	66	1	1	9	6	41	3	1	16	8
Down-weighting (weights: default = 29; 0 = excluded)	19	20	23	26	29	58	29	29	28	58	28	27	27	56	28	29	27	29	24	28	28	29	28	29	23	29	23	28	27	29	26	18	58	23	20	29	28	28	24	29	23	29	19	28	25	28	29	28	26	29	58	29	29	28	28	28	28	29	28	58	28	28	29	27	28	58	29	29	28	25	0	29	29	21	24	0	27	29	14	22
Jansen et al. 2002																																																																						0					0				
Lira et al. 2009																																																																						0					0				

	115	116	117	118	128	129	130	131	135	136	138	139	142	143	144	145	146	147	148	152	154	157	162	163	164	165	166	170	172	173	175	176	177	178	179	180	181	182	183	184	185	189	196	197	198	199	200	201	209	211	212	213	215	216	218	221	222	223	224	225	226	228	229	232	233	234	235	236	237	239	241	242	244	249	250	252	254	256	257
Position: 15.494-15.740	602	603	604	605	615	616	617	618	622	623	625	626	629	630	631	632	633	634	635	639	641	644	649	650	651	652	653	657	659	660	662	663	664	665	666	667	668	669	670	671	672	676	683		684	685	686	687	695	697		698	700	701	703	706	707	708	709	710	711	713		716	717	718	719	720	721	723	725	726	728	733	734		737	739	740
Nucleotide sequence of the reference X79547; *Xu and Arnason, 1994* (Insertions red colored)	C	T	G	C	A	A	T	A	A	T	T	A	T	A	A	T	A	T	C	T	T	T	A	A	G	T	A	T	T	A	T	A	T	T	G	A	T	C	G	T	G	A	C		C	A	A	G	A	T		T	C	A	T	A	C	A	C	G	C	T		C	A	C	A	G	C	C	T	G	T	G	A		T	A	A
Sites with deletions																																																																																
Monomorphic sites																																																																																
Polymorphic sites 2 nucleotides																																																																																
Polymorphic sites 3 nucleotides																																																																																
Max number of mutations at character	21	11	38	1	4	2	11	2	1	3	1	8	1	1	3	4	2	1	18	1	1	2	14	38	4	2	2	2	14	1	1	1	1	1	12	10	1	1	1	3	2	1	11	1	4	1	1	1	1	1	1	1	1	1	27	2	1	4	8	1	3	1	1	3	2	16	3	7	1	2	1	13	5	3	1	1	5	1	12
Down-weighting (weights: default = 29; 0 = excluded)	9	19	0	29	26	28	19	28	29	27	29	22	29	29	27	26	28	29	12	29	29	28	16	0	26	28	28	28	16	29	29	29	29	29	18	20	29	29	29	27	28	29	19	58	26	29	29	29	29	29	29	29	29	29	3	28	29	26	22	29	27	29	58	27	28	14	27	23	29	28	29	17	25	27	58	58	25	29	18
Jansen et al. 2002																									0					1,45																																																1,45		
Lira et al. 2009																									0					2,9																																																2,9		
