# Supplementary material for: Origin and History of Mitochondrial DNA Lineages in Domestic Horses
Source: PLoS One. 2010 Dec 20;5(12):e15311. doi: 10.1371/journal.pone.0015311 (PMC3004868; doi:10.1371/journal.pone.0015311)
Supplement: Supplementary file 9 [file pone.0015311.s005.doc]

		A	B	B1	D	D2	D2d	D2e	D3	E	F	G1	G3	Gx4	H	H1	H1a	I	I1	K	K2	K2a	K2b	K2b1	K3	K3a	K3b	X2	X2a	X2b	X3	X3c	X3c1	X4a	X5	X7a	X7a1
CHINA, MONGOLIA	*Debao*	X			X	X			X	X																		X	X							X	
	Guan Mountain	X				X				X											X		X					X								X	
	Guanzhong			X																X																	
	Mongolian	X		X		X				X											X	X	X		X			X		X			X				
	Taishu					X																						X									
	Tibetan									X			X	X							X							X		X	X		X				
	Wenshan																																				
	XiNiHe																																X				
	Dali							X																													
	Yunnan																																				
WEST AND SOUTH SIBERIA	*Akhal Teke*	X		X		X			X			X		X				X			X		X					X		X			X				X
	Caspian				X	X					X							X										X		X	X						
	Vyatskaya	X				X			X	X															X			X									
	Tuva	X							X									X			X	X											X	X		X	
NORTH WEST EUROPE	*Duelmener*								X																			X									
	Exmoor			X							X																										
	Gotland								X		X																				X						
	Icelandic			X							X												X					X									
	Norwegian Fjord			X					X														X		X			X									
	Shetland												X										X							X			X				
	Welsh							X																				X									
MEDITERANEAN AREA	*Anatolian*								X	X	X							X								X		X		X							
	Giara																																				
	Mallorquina																													X							
	Pindos			X					X		X												X					X		X				X			
	Sanfratellano	X		X					X		X									X								X					X	X			
	Sicilian Oriental Purebred																											X									
	Sicilian Indigenous			X		X			X										X					X				X		X			X	X			X
	Skyros										X										X												X				
EAST EUROPE	*Hucul*	X									X														X					X			X				
	Konik	X				X																															
IBERIAN PENINSULA	*Asturcon*								X		X												X					X					X				
	Cartujano																	X										X		X			X				
	Garrano			X											X													X			X		X				
	Losino							X	X									X										X					X				
	Lusitano																X						X					X		X		X					
	Marismeno								X							X												X			X						
	Merens								X		X							X										X									
	Pottoka								X																			X		X							
	Sorraia																													X	X		X				
AFRICA	*Arabian*	X	X	X	X	X			X									X	X	X	X		X		X	X		X		X	X		X				X
	Barb	X			X				X					X				X					X		X			X									
	Egyptian	X								X										X					X								X				
	Fulani								X																			X							X		
NORTH EAST SIBERIA	*Yakut*	X				X															X					X	X	X			X						
KOREA	*Cheju*	X					X			X	X							X			X				X						X		X	X			
