# Supplementary material for: Origin and History of Mitochondrial DNA Lineages in Domestic Horses
Source: PLoS One. 2010 Dec 20;5(12):e15311. doi: 10.1371/journal.pone.0015311 (PMC3004868; doi:10.1371/journal.pone.0015311)
Supplement: Supplementary file 10 [file pone.0015311.s008.doc]

| | Tamura & Nei; Permut.: 10,000 | *LATE PLEISTOCENE* | | | *COPPER AGE* | | *BRONZE AGE* | | | | *IRON AGE* | | | *MEDIEVAL* |
|---|---|---|---|---|---|---|---|---|---|---|---|---|---|---|
| | | *Alaska* | *North East Siberia* | *Europe* | *Europe / Asia Minor / Armenia* | *Iberian Peninsula* | *China* | *West Siberia* | *Europe / Armenia* | *Iberian Peninsula* | *China* | *West / SouthSiberia, Kazakhstan* | *Europe* | *Europe* |
| ***LATE PLEISTOCENE*** | *Alaska* | / | x | x | x | x | x | x | x | x | x | x | x | x |
| | *North East Siberia* | 0.223 | / | | x | x | | | x | x | | | | |
| | *Europe* | 0.293 | 0.016 | / | | x | x | | | | | | | |
| ***COPPER AGE*** | *Europe / Asia Minor / Armenia* | 0.319 | 0.088 | 0.042 | / | x | x | | | x | x | x | x | x |
| | *Iberian Peninsula* | 0.481 | 0.172 | 0.136 | 0.162 | / | x | x | x | | x | x | x | x |
| ***BRONZE AGE*** | *China* | 0.337 | 0.107 | 0.175 | 0.220 | 0.433 | / | | x | x | | | | |
| | *West Siberia* | 0.201 | 0.019 | -0.006 | 0.018 | 0.176 | 0.012 | / | | x | | | | |
| | *Europe / Armenia* | 0.224 | 0.050 | 0.003 | 0.007 | 0.145 | 0.107 | -0.057 | / | x | | | | |
| | *Iberian Peninsula* | 0.389 | 0.125 | 0.051 | 0.098 | -0.009 | 0.281 | 0.093 | 0.071 | / | x | x | x | x |
| ***IRON AGE*** | *China* | 0.190 | 0.033 | 0.032 | 0.057 | 0.129 | 0.058 | -0.035 | 0.001 | 0.107 | / | | x | |
| | *West / South Siberia, Kazakhstan* | 0.192 | 0.017 | 0.008 | 0.065 | 0.118 | 0.054 | 0.002 | 0.003 | 0.105 | 0.019 | / | | |
| | *Europe* | 0.169 | 0.050 | -0.005 | 0.074 | 0.183 | 0.083 | 0.006 | 0.005 | 0.163 | 0.059 | -0.005 | / | |
| ***ME-DIEVAL*** | *Europe* | 0.214 | 0.034 | 0.024 | 0.065 | 0.132 | 0.026 | -0.014 | -0.016 | 0.108 | 0.022 | -0.011 | 0.000 | / |
