# Supplementary material for: Origin and History of Mitochondrial DNA Lineages in Domestic Horses
Source: PLoS One. 2010 Dec 20;5(12):e15311. doi: 10.1371/journal.pone.0015311 (PMC3004868; doi:10.1371/journal.pone.0015311)
Supplement: Supplementary file 11 [file pone.0015311.s009.doc]

	Permutation: 10.000	LATE PLEISTOCENE			COPPER AGE		BRONZE AGE				IRON AGE			MEDIEVAL
		Alaska	*North East Siberia*	*Europe*	*Europe / Asia Minor / Armenia*	*Iberian Peninsula*	*China*	*West Siberia*	*Europe / Armenia*	*Iberian Peninsula*	*China*	*West / South Siberia, Kazakhstan*	*Europe*	*Europe*
LATE PLEISTOCENE	*Alaska*	**4.085**	5.130	4.998	5.704	4.911	5.650	5.608	6.175	5.392	5.538	6.211	7.042	6.741
	North East Siberia	1.133	**3.911**	3.538	4.188	3.188	4.089	4.443	4.881	3.829	4.503	4.902	5.907	5.240
	Europe	1.449	0.075	**3.014**	3.527	2.432	3.797	3.910	4.246	3.103	4.103	4.477	5.261	4.814
COPPER AGE	*Europe / Asia Minor / Armenia*	1.800	0.371	0.158	**3.723**	2.995	4.544	4.350	4.585	3.615	4.537	5.089	6.008	5.351
	Iberian Peninsula	2.259	0.622	0.315	0.523	**1.221**	3.560	3.641	3.937	2.096	3.713	4.184	5.241	4.559
BRONZE AGE	*China*	1.965	0.491	0.647	1.040	1.307	**3.286**	4.197	5.046	4.194	4.433	4.980	6.182	5.095
	West Siberia	1.139	0.062	-0.024	0.062	0.605	0.128	**4.853**	4.847	4.117	4.636	5.301	6.254	5.454
	Europe / Armenia	1.439	0.232	0.046	0.030	0.634	0.710	-0.272	**5.387**	4.330	5.057	5.548	6.486	5.677
	Iberian Peninsula	1.904	0.429	0.151	0.309	0.041	1.107	0.246	0.192	**2.890**	4.271	4.729	5.729	4.994
IRON AGE	*China*	1.119	0.171	0.219	0.300	0.726	0.413	-0.167	-0.013	0.449	**4.753**	5.302	6.376	5.551
	West / South Siberia, Kazakhstan	1.336	0.115	0.137	0.396	0.741	0.505	0.042	0.022	0.452	0.093	**5.664**	6.527	5.830
	Europe	1.187	0.138	-0.059	0.334	0.817	0.726	0.014	-0.021	0.471	0.186	-0.119	**7.627**	6.793
ME-DIEVAL	*Europe*	1.640	0.225	0.248	0.430	0.889	0.392	-0.032	-0.076	0.490	0.115	-0.062	-0.080	**6.119**
