# Supplementary material for: Origin and History of Mitochondrial DNA Lineages in Domestic Horses
Source: PLoS One. 2010 Dec 20;5(12):e15311. doi: 10.1371/journal.pone.0015311 (PMC3004868; doi:10.1371/journal.pone.0015311)
Supplement: Supplementary file 12 [file pone.0015311.s012.doc]

	Sequence section of HVR I	Name	Sequence 5`to 3`	bp	Length
Primer set I	15.468-15.490	K1F	GAT TTC TTC CCC TAA ACG ACA AC	23	108
	15.551-15.575	K1R	CTG ACA TGC AAT ATC TTA TGA ATG G	25	
	15.546-15.566	K2F	CCC ACC TGA CAT GCA ATA TCT	21	144
	15.670-15.689	K2R	GTG CAT ACC CCA TCC AAG TC	20	
	15.663-15.681	K3F	ATT RAT CGT GCA TAC CCC A	19	134
	15.779-15.796	K3R	GTG TCC CAA TCC TCG CTC	18	
	15.752-15.769	K4F	CGG GAA ATC AGC AAC CCT	18	150
	15.882-15.902	K4R	GCC CAT TCT TTC CCC TTA AAT	21	
	15.848-15.866	K5F	GGT TCT TTC TTC AGG GCC A	19	155
	15.981-16.003	K5R	TAT TTG GGG ATG CTA TGA CTC AG	23	
	15.963-15.988	K6F	GCA TTT GGT ATY TTT TTA TAT TTG GG	26	127
	16.071-16.090	K6R	TCC GCA TCA RCA ACC ATA AG	20	
Primer set II	15.473-15.492	HP1F	CTT CCC CTA AAC GAC AAC AA	20	116
	15.570-15.588	HP1R	GAA TGG CCT ATG TAC RTC G	19	
	15.528-15.547	HP2F	CCC CCA YAT AAC ACY ATA CC	20	153
	15.662-15.680	HP2R	TAT TRA TCG TGC ATA CCC C	19	
	15.604-15.622	HP3F	GCC CCA TGA ATA ATA AGC A	19	178
	15.763-15.781	HP3R	CAA CCC TCY CAA CTA CGT G	19	
	15.714-15.732	HP4F	ATC ACA RCC CAT GTT CCA C	19	153
	15.848-15.867	HP4R	GGT TCT TTC TTC AGG GCC AT	20	
	15.808-15.825	HP5F	AAA CGT GGG GGT TTC TAC	18	136
	15.927-15.944	HP5R	TAA TCA GCC CAT GCT CAC	18	
	15.882-15.899	HP6F	GCC CAT TCT TTC CCC TTA	18	137
	16.001-16.019	HP6R	CAG CTA TGG CCG TCA AAG	18	
	15.965-15.987	HP7F	ATT TGG TAT YTT TTT ATA TTT GG	23	116
	16.065-16.081	HP7R	TAT YCC TCC GCA TCA GC	17	
