# Supplementary material for: Origin and History of Mitochondrial DNA Lineages in Domestic Horses
Source: PLoS One. 2010 Dec 20;5(12):e15311. doi: 10.1371/journal.pone.0015311 (PMC3004868; doi:10.1371/journal.pone.0015311)
Supplement: Supplementary file 13 [file pone.0015311.s013.doc]

Horse breed	*No. of Haplogroups / No.of samples*	*Typical geographic region of the breed*	*Acession No. / Haplogroup / Haplotype*
Akhal Teke	10/37	West Siberia	DQ327964 (A), DQ327960 (A), DQ327956 (A), AY246175 (B1), AY246179 (B1), DQ327952 (B1), EU093059-60 (D2), EU093051 (D2), EU093052-53 (D3), EU093061 (D3), EU093054 (G1), DQ327957 (Gx4), EU093055-56 (Gx4), EU093057 (G-group), DQ327959 (G-roup), DQ327958 (I), EU093062 (I), EU093049 (I), AY246174 (K2b), EU093063 (K2b), DQ327962-63 (X2), DQ327965-67 (X2b), DQ327961 (X2b), DQ327953 (X2b), DQ327954-55 (X3c1), DQ327951-52 (X3c1), EU093058 (X3c1), EU093050 (X3-group), AY246177 (X7a1)
Anatolian	7/17	Asia Minor	AF481243 (D3), AF481237 (D3), AF481233 (D3), AF481232 (E), AF481238 (E), AF481244 (F), AF481241-42 (F), AF481245 (I), AF481236 (I), AY246176 (K2), AY246178 (K2), AF481239 (K3a), AF481240 (X2), AF481234 (X2), AF481235 (X2b), AF481246 (X2-group)
Arabian	10/99	Africa	EF437554 (A), AJ413645-46 (A), AJ413642-43 (A), AJ413639 (A), AF132576-77 (A), AF465997 (A-linked), AF132589 (A-linked), AF132589 (A-linked), AF132588 (B), AF132588 (B), EF437555 (B1), AJ413652 (B1), AJ413654 (B1), AJ413650 (B1), EF437565 (D), AF466003 (D2), AF132587 (D2), AY246180-85 (D3), EF437556 (D3), AJ413655 (D3), AJ413648 (D3), AJ413640 (D3), AF132591 (D3), AF132591 (D3), EF437566 (I), EF437558 (I), AF466000 (I), AJ413647 (I), AJ413644 (I), AJ413634 (I), AJ413636 (I), AJ413631-32 (I), AF132594 (I), AF132585-86 (I), AF466001 (I-group), AF132584 (I1), AF132584 (I1), AF132575 (K), AF132575 (K), EF437560 (K2), EF437557 (K2), AJ413633 (K2), AJ413635 (K2), AJ413630 (K2), AF132571 (K2), AF132571 (K2), EF437561 (K2b), AJ413637 (K2b), AF132593 (K2b), AF132572 (K2b), AF132593 (K2b), AF132572 (K2b), EF437562 (K3), AF465999 (K3), AJ413653 (K3), AJ413651 (K3), AJ413629 (K3), AF132574 (K3), AF132568 (K3), AF132574 (K3), AF132568 (K3), AF132573 (K3-group), AF132573 (K3-group), AF466004 (K3a), AJ413641 (K3-group), AF466005 (K3-group), EF437559 (X2), EF437553 (X2), AJ413657 (X2), AF132582-83 (X2), AF465998 (X2-group), EF437563 (X2b), AF132570 (X2b), AF132570 (X2b), AF466002 (X3-group), AF132569 (X3-group), AF132569 (X3-group), AJ413656 (X3), AY246184 (X3c1), EF437564 (X3c1), AF465996 (X3c1), AJ413638 (X3c1), AF132578 (X3c1), AF132592 (X3c1), AF132578 (X3c1), AF132592 (X3c1), AF132590 (X7a1), AF132590 (X7a1)
Asturcon	5/12	Iberian Peninsula	AY519873 (D3), AY519878 (D3-group), AY519874 (F), AY519882 (K2b), AY519872 (K2b), AY519879-81 (X2), AY519877 (X2), AY519875 (X2), AY519876 (X3c1), AY519871 (X3c1)
Barb	8/37	Africa	EF686043-44 (A), EF686030 (A), EF686028 (A), EF686025-26 (A), AJ413661 (A), EF686042(D), EF686035 (D), EF686022 (D), AJ413671 (F-group), AJ413664 (Gx4), AJ413665 (I), AJ413663 (K2b), AJ413669 (K3), EF686045 (X2), EF686040-41 (X2), EF686038 (X2), EF686031-34 (X2), EF686029 (X2), EF686027 (X2), EF686023-24 (X2), EF686021 (X2), AJ413670 (X2), AJ413667-68 (X2), AJ413662 (X2), AJ413658-60 (X2), AJ413666 (X2-group)
Cartujano	3/8	Iberian Peninsula	AY519903 (I), AY519905 (I), AY519899-900 (I), AY519906 (X2), AY519901 (X2), AY519898 (X2b), AY519904 (X3c1)
Caspian	5/13	West Siberia	AY246195 (D), AJ413726-29 (D2), AJ413730 (F), AY246198 (I), AJ413724-25 (X2), AY246197 (X2-group), AY246195 (X2b), AY246199 (X2b), AY246200 (X3)
Cheju	10/25	Korea	AF354440 (A), AF354429 (D2d), AY246205 (E), AY246203 (E), AF354427 (E), AY246202 (F), AF354437 (F), AY246208 (G-group), AF354425 (G-group), AY246204 (I), AY246207 (I), AF354441 (I), AF354430 (I), AF354428 (I), AY246206 (K2), AF354434-35 (K2), AF354439 (K2), AY246201 (K3), AF354433 (K3), AF354436 (K3-group), AF354431 (X3), AF354432 (X3c1), AF354438 (X3-group), AF354426 (X4a)
Debao	7/24	China	EF686039 (A), EF686036-37 (A), EU939445 (A), FJ392576 (A-linked), FJ392578 (D), FJ392572 (D2-group), FJ392575 (D2), FJ392563 (D2), FJ392566 (D), FJ392567 (E), FJ392571 (E), EU826536 (E), FJ392569 (E-group),FJ392574 (G-group), FJ392580 (X2), FJ392573 (X2), FJ392570 (X2), FJ392568 (X2), FJ392562 (X2), FJ392579 (X2a), FJ392565 (X3-group), FJ392564 (X3-group), FJ392577 (X7a)
Duelmener	2/10	Central Europe	AJ413673 (D3), AJ413677-81 (X2), AJ413674 (X2), AJ413672 (X2), AJ413675-76 (X2-group)
Egyptian	7/7	North Africa	AF481273 (A), AF481275 (E), AF481277 (I-group), AF481278 (K), AF481274 (K3), AF481276 (X2-group), AF481272 (X3c1)
Exmoor	5/17	North Europe	AF072992-93 (B1), AJ413682-83 (B1), AJ413686 (B1), AJ413688-91 (B1), AJ413684-85 (D2e) AJ413687 (D2e), AY246219 (F), AY246223 (F), AY246221 (I-group), AY246224 (I-group), AY246220 (X3-group)
Fulani	3/9	Africa	AF481287 (D3), AF481283-84 (D3), AF481288-90 (X2), AF481286 (X2), AF481285 (X5), AF481281 (X5-group)
Garrano	5/5	Iberian Peninsula	AY246232 (B1), AY246235 (H), AY246234 (K2), AY246233 (X2), AY246231 (X3)
Giara	1/2	Mediterranean	AY462429-30 (X3c1)
Gotland	3/3	North Europe	AY575107 (D3), AY575105 (F), AY575106 (X3)
Guan Mountain	6/10	China	DQ327846 (A), DQ327843 (D2), DQ327838 (E), DQ327845 (E), DQ327844 (K2), DQ327839 (K2b), DQ327840-42 (X2), DQ327847 (X7a)
Guanzhong	2/2	China	AY136786 (B1), AY136785 (K)
Hucul	5/11	East Europe	EU093066 (A), AY575113 (A), EU093070 (F), EU093067-68 (F), EU093069 (K3), EU093072 (K3), EU093064-65 (K3), AY575114 (X2b), EU093071 (X3c1)
Icelandic	4/6	North Europe	AJ413721 (B1), AJ413723 (B1), AJ413722 (F), AJ413720 (G-group), AJ413719 (K2b), AJ413717 (K2b)
Konik	3/5	East Europe	AJ413734 (A), AJ413733 (D2), AJ413735 (X3-group), AJ413731 (X3-group), AJ413732 (X3-group)
Losino	5/10	Iberian Peninsula	AY519930 (D2e), AY519931 (D3), AY519925 (D3), AY519928-29 (I), AY519924 (X2), AY519933 (X3-group), AY519926 (X3-group), AY519932 (X3-group), AY519927 (X3c1)
Lusitano	5/10	Iberian Peninsula	AF466008 (F-group), AY525095 (H1a), AY525094 (K2b), AY246247 (X2), AY246243 (X2b), AY246246 (X2b), AY246245 (X3c), AY246244 (X3c), AY246242 (X3c), AY525096 (X3c)
Mallorquina	2/2	Mediterranean	AF466013 (A-linked), AF466014 (X2)
Marismeno	4/12	Iberian Peninsula	AY519941 (D3), AY519937-38 (D3), AY519934 (D3), AY519945 (H1), AY519943 (X2), AY519939-40 (X2), AY519935-36 (X2), AY519942 (X3), AY519944 (X3)
Merens	5/10	Iberian Peninsula	AY519957 (B-group), AY519949 (D3), AY519954 (F), AY519951 (F), AY519948 (F), AY519947 (I), AY519946 (X2-group), AY519956 (X2), AY519952-53 (X2)
Mongolian	10/20	Mongolia	AB329592 (A), DQ297628 (A-linked), AB329604 (B2), DQ297622 (D2), DQ297629 (D3), AB329606 (E), DQ297625 (I-group), DQ297633 (K-linked), AB329625 (K2a), AF072996 (K2b), DQ297623 (K3), DQ297632 (K3), AB329622-23 (K3), AB329628 (K3), DQ297624 (X2), AB329618 (X2-group), DQ297626 (X2b), DQ297627 (X3-group), DQ297631 (X3c1)
Norwegian Fjord	6/11	North Europe	AJ413701 (B1), AJ413703 (B1), AJ413700 (B-group), AJ413696-98 (D3), AJ413702 (I-group), AJ413696 (K2), AJ413699 (K2), AJ413694 (K3), AJ413704 (X2)
Pindos	6/7	Mediterranean	EF433386 (B1), EF433384 (D3), EF433387 (F), EF433382 (K2), EF433385 (X2), EF433383 (X2b), EF433388 (X4a)
Pottoka	3/13	Iberian Peninsula	AY519968 (D3), AY519959 (X1-group), AY519963 (X1-group), AY519961 (X1-group), AY519960 (X1-group), AY519958 (X2-group), AY519970 (X2), AY519967 (X2-group), AY519965 (X2b), AY519969 (X2b), AY519962 (X2b), AY519964 (X2-group), AY519966 (X2-group)
Przewalskii	1/2	China/Mongolia	AF014409 (X3-group), AF072995 (X3-group)
Sanfratellano	8/10	Mediterranean	EU750724 (A), EU750722 (A), EU750725 (B1), EU831238 (D3), EU831234 (F), EU750723 (K), EU750730 (X2), EU750717 (X3c1), EU750726 (X4a), EU569297 (X4-group)
Shetland	5/18	North Europe	AF481295-97 (B-group), AJ413920 (G-group), AY246257 (G3), AY246253 (G3), AF481294 (G3), AY246256 (K2), AY246258 (K2), AF481298-300 (K2), AY246254-55 (X2-group), AF481301-04 (X2b), AJ413919 (X3c1)
Sicilian Oriental Purebred	1/1	Mediterranean	EU750729 (X2)
Sicilian Indigenous	10/13	Mediterranean	EU750716 (B1), EU750731 (D2), EU750718 (D3), EU604817 (F), EU750719 (I1), EU604816 (K2b1), AJ413718 (X2), EU750728 (X2), EU604816 (X2-group), EU750727 (X2b), EU831237 (X3c1), EU831239 (X4a), EU750721 (X7a1)
Skyros	3/5	Mediterranean	EF433398-99 (F), EF433395 (F), EF433400 (K2), EF433397 (X3c1)
Sorraia	2/27	Iberian Peninsula	DQ339575 (X2-group), AY246261-63 (X3), AF447765 (X3), AJ413904-09 (X3), AJ413917 (X3), AJ413910-16 (X3c1), AJ413918 (X3c1), AJ413901-03 (X3c1), AF447764 (X3c1), AY246259-60 (X3c1), AY246264 (X3c1)
Taishu	2/2	Japan	AB329588 (D2), AB329614 (X2)
Tibetan	6/16	Tibet	DQ986469 (A-linked), DQ986475 (A-linked), DQ986467 (E), DQ986474 (E), DQ986479 (E), DQ986466 (Gx4), DQ986471-72 (G3), DQ986473 (K2), DQ986477 (K2), DQ986464 (X2), DQ986468 (X2-group), DQ986476 (X2b), EF597513 (X3c1), DQ986470 (X3c1), DQ986478 (X3)
Tuva	8/10	South Siberia	AF481324 (A), AF481328 (D3), AF481332 (I), AF481325 (K2), AF481330 (K2a), AF481333 (X2-group), AF481331 (X2-group), AF481334 (X3c1), AF481326 (X4a), AF481327 (X7a)
Vyatskaya	6/18	West Siberia	DQ328033 (A), DQ328028 (A), DQ328023 (A), DQ328027 (D2), DQ328022 (D2), DQ328035 (D3), DQ328032 (D3), DQ328029 (D3), DQ328024 (D3), DQ328025 (E), DQ328030 (E), DQ328034 (K3), DQ328036 (X2), DQ328020 (X2), DQ328021 (X2-group), DQ328031 (X2-group), DQ328026 (X2-group), DQ328037 (X2-group)
Welsh	1/1	North Europe	AJ413926 (D2e)
Wenshan	2/2	China	FJ502837 (E-group), FJ502836 (X2)
XiNiHe	1/1	China	DQ297638 (X3c1)
Dali	2/6	China	FJ502834 (D2e), DQ328051 (X2), DQ328049 (X2), DQ328045-46 (X2), DQ328038 (X2)
Yakut	5/15	North East Siberia	DQ328055 (A), DQ328043 (A), DQ328041 (A), DQ328039 (A), DQ328053 (A-linked), DQ328057 (D2), DQ328054 (D2), DQ328042 (K2), DQ328050 (K3b), DQ328052 (K3a), DQ328044 (K3-group), DQ328056 (X3), DQ328047-48 (X3), DQ328040 (X3)
Yunnan	1/1	China	AF014417 (X2)
